# The athletic gut microbiota

**DOI:** 10.1186/s12970-020-00353-w

**Published:** 2020-05-12

**Authors:** Alex E. Mohr, Ralf Jäger, Katie C. Carpenter, Chad M. Kerksick, Martin Purpura, Jeremy R. Townsend, Nicholas P. West, Katherine Black, Michael Gleeson, David B. Pyne, Shawn D. Wells, Shawn M. Arent, Richard B. Kreider, Bill I. Campbell, Laurent Bannock, Jonathan Scheiman, Craig J. Wissent, Marco Pane, Douglas S. Kalman, Jamie N. Pugh, Carmen P. Ortega-Santos, Jessica A. ter Haar, Paul J. Arciero, Jose Antonio

**Affiliations:** 1 College of Health Solutions, Arizona State University, Phoenix, AZ USA; 2 Increnovo LLC, Milwaukee, WI 53202 USA; 3 Isagenix International LLC, Gilbert, AZ USA; 4 Exercise and Performance Nutrition Laboratory, School of Health Sciences, Lindenwood University, St. Charles, MO USA; 5 Exercise and Nutrition Science Graduate Program, Lipscomb University, Nashville, TN 37204 USA; 6 School of Medical Research and Menzies Health Institute of QLD, Griffith Health, Griffith University, Southport, Australia; 7 Department of Human Nutrition, University of Otago, Dunedin, New Zealand; 8 School of Sport, Exercise and Health Sciences, Loughborough University, Loughborough, UK; 9 Research Institute for Sport and Exercise, University of Canberra, Canberra, ACT 2617 Australia; 10 WGI, Lewisville, TX USA; 11 Department of Exercise Science, Arnold School of Public Health, University of South Carolina, Columbia, SC USA; 12 Exercise & Sport Nutrition Lab, Human Clinical Research Facility, Department of Health & Kinesiology, Texas A&M University, College Station, TX 77843-4253 USA; 13 Performance & Physique Enhancement Laboratory, University of South Florida, Tampa, FL USA; 14 Institute of Performance Nutrition, London, UK; 15 Fitbiomics, Inc, New York, NY USA; 16 Jamieson Wellness Inc., 4025 Rhodes Drive, Windsor, Ontario N8W 5B5 Canada; 17 Bioloab Research, Via E. Mattei 3, 28100 Novara, Italy; 18 Scientific Affairs, Nutrasource Diagnostics, Inc. Guelph, Guelph, Ontario Canada; 19 Research Institute for Sport and Exercise Sciences, Liverpool John Moores University, Tom Reilly Building, Byrom St Campus, Liverpool, L3 3AF UK; 20 International Probiotic Association, Los Angeles, CA USA; 21 Health and Human Physiological Sciences Department, Skidmore College, Saratoga Springs, NY USA; 22 Exercise and Sport Science, Nova Southeastern University, Davie, FL USA

**Keywords:** Athletes, Gut microbiome, Microbial ecology, Gut health, Sports nutrition, Sport performance, Exercise, Physical activity, Metagenome, Short-chain fatty acids

## Abstract

The microorganisms in the gastrointestinal tract play a significant role in nutrient uptake, vitamin synthesis, energy harvest, inflammatory modulation, and host immune response, collectively contributing to human health. Important factors such as age, birth method, antibiotic use, and diet have been established as formative factors that shape the gut microbiota. Yet, less described is the role that exercise plays, particularly how associated factors and stressors, such as sport/exercise-specific diet, environment, and their interactions, may influence the gut microbiota. In particular, high-level athletes offer remarkable physiology and metabolism (including muscular strength/power, aerobic capacity, energy expenditure, and heat production) compared to sedentary individuals, and provide unique insight in gut microbiota research. In addition, the gut microbiota with its ability to harvest energy, modulate the immune system, and influence gastrointestinal health, likely plays an important role in athlete health, wellbeing, and sports performance. Therefore, understanding the mechanisms in which the gut microbiota could play in the role of influencing athletic performance is of considerable interest to athletes who work to improve their results in competition as well as reduce recovery time during training. Ultimately this research is expected to extend beyond athletics as understanding optimal fitness has applications for overall health and wellness in larger communities. Therefore, the purpose of this narrative review is to summarize current knowledge of the athletic gut microbiota and the factors that shape it. Exercise, associated dietary factors, and the athletic classification promote a more “health-associated” gut microbiota. Such features include a higher abundance of health-promoting bacterial species, increased microbial diversity, functional metabolic capacity, and microbial-associated metabolites, stimulation of bacterial abundance that can modulate mucosal immunity, and improved gastrointestinal barrier function.

## Introduction

The human gut microbiota contains thousands of different bacterial taxa as well as various archaea, eukaryotic microbes and viruses, more than three million genes, and harbors an enormous metabolic capacity [1, 2]. The microorganisms in the gastrointestinal (GI) tract play a role in nutrient uptake, vitamin synthesis, energy harvest, inflammatory modulation, and host immune response [3, 4]. In turn, numerous intrinsic and extrinsic factors can affect the gut microbiota which results in a complex gut ecosystem that is highly dynamic and individual [5, 6]. Important factors such as age, birth delivery route, antibiotic use, and diet can shape the gut microbiota [7–10]. The role that exercise plays, in particular the associated factors and stressors, such as sport/exercise-specific diet [11], environment [12], and their interactions, on the gut microbiota have been less well described. Athletes, although diverse as a population given the wide variety of different types of exercise/fitness/athletic training, competition, dietary practices and attributes, exemplify these factors on a generally consistent and long-term basis.

High-level athletes possess remarkable physiological and metabolic adaptations (including muscular strength/power, aerobic capacity, energy expenditure, and heat production) and provide unique insight in gut microbiome research. In addition, the gut microbiota with its ability to harvest energy, modulate the immune system, and influence mucosal and brain health, is likely to play a significant role in athlete health, wellbeing, and sports performance [13–15]. The microbiota has an indirect influence on various indices of exercise performance, recovery, and patterns of illness, such as signaling through myokines and other cytokines, modulating activation of the hypothalamic–pituitary–adrenal axis, and affecting performance-associated metabolic pathways [13, 16–18]. Understanding the various roles the gut microbiota plays in relation to athletic performance is of great interest to athletes seeking to improve competition outcomes as well as reduce recovery time from training. Such knowledge may be of general benefit to further understanding of microbial contributions to human health and disease. Current research reports a higher abundance of health-promoting bacterial species and increased microbial diversity in athletes [13, 18, 19].

Given the increasing interest in exercise, associated dietary factors, and athletes as a population in relation to the gut microbiota, the purpose of this narrative review is to summarize current knowledge of the athletic gut microbiota and the factors that shape it. While differences likely exist among how the gut microbiota is affected by the different types of sport/athlete/fitness training regimens (e.g., resistance, interval, stretching/flexibility, endurance/aerobic, etc.), the primary aim is to provide a “state-of-the-art research” statement. Key topics covered include:

- How the athletic/exercise-associated gut microbiota differs in comparison to other populations.
- The effects of different types of exercise training on the gut microbiota.
- The effects of an ‘athletic diet’ on the gut microbiota.

### Influencing factors that shape the gut microbiota

Numerous factors such as age, genetics, drug use, stress, smoking, and diet can all affect the microbial composition of the gut, influencing a complex ecosystem that is highly dynamic and individual [5–7, 20]. For example, the manner in which we are born and raised can result in substantial differences in the composition of the gut microbiota. This outcome is related to the differences in exposure (or non-exposure) of bacteria in the birth canal during vaginal birth [10], being bottle fed or breastfed [21], living with a dog, cat, or close to farm animals [22], the number of antibiotic treatments administered [8], and environmental toxin exposure [23]. From birth until the age of about 3 years, an individual assembles their core of resident microbiota primarily dominated by gram-positive *Firmicutes* and gram-negative *Bacteroidetes* phyla, and this subsequent make-up is as unique as a set of fingerprints [24–26]. The gut microbiota is also essential for processing dietary components and appears to have a major role in shaping the immune system [27]. Not surprisingly, the role of the gut microbiota in determining host health and development of disease has been gaining clinical and community interest [9]. Altered gut microbiota composition and/or function is linked to a growing number of conditions from metabolic disorders to some brain-related dysfunctions [28, 29]. Individuals in a known disease state can have a significantly different gut microbiota composition compared with healthy individuals [30]. A common observation is increased species diversity and/or richness in the gut microbiota of healthy individuals. Although new research suggests gene content/diversity in the gut may be a better predictor of physiological states [31]. In addition, counterexamples exist as recent work links high gut microbial diversity with a longer colonic transit time and systemic circulation of potentially harmful protein degradation products [32]. On a compositional level, it could be that low diversity indicates poorer health, while high diversity does not always guarantee improved health. Thus, information about compositional diversity alone is not sufficient to assess the health of the microbiota (and the host). Although, from an ecological perspective, functional diversity may be a key factor in allowing an ecosystem to continue operating properly [33]. Resilience to both external and internal changes (with the ability to rapidly return to its baseline functional profile) is likely a key feature of a healthy gut microbiota’s ability to maintain itself [34].

In individuals without disease, “health-associated” microbiota is preferred to the term “healthy microbiota”, since gut microbial composition alone cannot predict any state of health or disease according to currently available research [30, 34]. It may turn out that many possible states of microbial composition are associated with health or indeed that a “health-associated” microbiota is more resilient and resistant to disruption [35]. It is also important to keep in consideration that gut microbiome composition is quite stable over time [36, 37]. For example, ~ 60% of microbiome composition was found to be stable up to a 5-year period in US adults [38]. In addition, while species composition varies tremendously from person to person, there is substantial functional redundancy at the metabolic pathway level [1, 2, 39]. Therefore, looking at metagenomic functions rather than taxonomy alone may provide for a better appreciation of the true gut microbiome metabolic activity and the impact of microbial functions on human physiology [40, 41]. Physical activity has been an area of growing interest in gut microbiota research and appears to promote a health-associated microbial community and increased metabolic functional potential. This work includes identifying the impact that varying and diverse athletic and physical activity regimens exert on the gut microbiota.

Physical activity focused gut microbiota research is quite new and enabled by dramatic increases in scale and scope due to advances in DNA-sequencing technologies coupled with computational methods needed given the incredible information density of the microbiota [42–44]. Data are obtained predominantly from next-generation sequencing in three forms: A) ribosomal RNA (rRNA) gene sequence surveys that provide a view of microbiome membership, B) metagenomic data used to portray functional potential, and, C) metatranscriptomic data to describe active gene expression (for a review, readers are directed to [42]). Currently, 16 rRNA gene surveys are the most commonly used as they are substantially more economical and therefore scale to larger projects [44]. However, this technique is limited by short read lengths obtained, sequencing errors, and differences arising from the different regions chosen (e.g., hypervariable region V3 vs V4) [45]. 16 rRNA sequencing also has limited resolution and lower sensitivity compared to whole-community shotgun metagenomic analysis, such as characterization down to the genus level with minimal capability of species-level detection [46]. Therefore, shotgun metagenomics is displacing 16S rRNA amplicon analysis because of its expanded taxonomic range and strain-level resolution [42].

Analyses used to interpret large data sets generated from these high-throughput sequencing techniques commonly include measures of biological diversity. Many of the studies included in this review measured alpha diversity which represents diversity within a sample. In calculating alpha diversity, various metrics (e.g., Shannon index, Chao1) consider the number of unique operational taxonomic units (OTUs), termed ‘richness’, and their relative abundance, termed ‘evenness’. Also often used is beta-diversity, a measurement of how different the communities are between samples. Beta-diversity metrics are quantitative (e.g., weighted UniFrac), when considering samples phylogeny, and qualitative (e.g., uniweighted UniFrac) when only evaluating the presence/absence of samples [47]. In addition, other ‘omic’ techniques, such as metabolomics, are being integrated with these data to provide deeper insights into host metabolism and health. Metabolomics uses high throughput techniques to characterize and quantify small molecules in several biofluids (urine, serum, plasma, feces, saliva), revealing a unique metabolic signature [48]. As a complement to sequencing-based approaches, the use of metabolomics (particularly from feces) is encouraged as it offers a ‘functional’ readout of the microbiome providing data on the metabolic interplay between the host, diet, exercise, and the gut microbiota [49, 50].

While the aforementioned techniques have allowed for a rapid increase in gut microbiota research, the variation in microbial analysis across different studies can make comparing/contrasting study findings difficult. Indeed, variation in profiling techniques (e.g., sequencing strategy, platform, variable regions, sequencing depth, etc.) may act as a confounding variable resulting in divergent findings due entirely to laboratory techniques rather than treatment [51]. Furthermore, many gut microbiota studies may be underpowered, and scientists may not be controlling for important confounding variables such as diet, gender, ethnicity, GI problems, antibiotics, etc. As the investigation of the athletic microbiota is a newer field of research, the intention of this review is to provide a broad overview of the current state of the literature. For future, more specific reviews, deeper discussions of methodological nuances are warranted.

### The athlete/exercise-associated gut microbiota

Establishing consistent relationships across studies in physically active groups, such as athletes has been problematic. Beyond the obvious methodological differences such as sample preparation techniques, DNA sequencing, bioinformatics tools, and reference databases [52–55], there is also large variation across exercise/athletic regimens. Moreover, confounding factors including training history, level of physical fitness, training environment, and dietary intake all have the potential to affect study outcomes substantially, and make detecting differences due to exercise/athletic regimens on the gut microbiota difficult to ascertain. Therefore, when comparing these individuals within or across various exercise/athletic disciplines and classifications, these factors should be accounted for and reported by investigators. While the current body of this comparative-type research is mixed and more limited (see Table 1), collectively it provides important insight and highlights key areas of future study.

**Table 1:** Athlete/exercise-associated gut microbiota in comparison to other populations or across athletic classification: Characteristics of included articles (by publication date)

Authors, year, country	Subjects	Sex and age	Study design and gut microbiome analysis	Diet and/or exercise	Key outcome(s)
1. Clarke et al., 2014, Ireland [19]	Professional male rugby athletes (*n* = 40) and healthy size, age and sex matched controls (*n* = 46)	86 Males 23–35 years	Cross-sectional, observational 16S rRNA gene amplification of the V4 region	Diet recorded by a 187-food item FFQ^a^. Physical activity levels assessed by EPIC^b^-Norfolk questionnaire.	• Athletes had a higher GM^c^ diversity compared to controls. • Athletes in low BMI^d^ (< 25 kg/m^2^) group had higher proportions of genus *Akkermansia* compared with high BMI (> 28 kg/m^2^) group.
2. Bressa et al., 2017, Spain [56]	Healthy premenopausal women; active defined by WHO^e^ (*n* = 19) and sedentary (*n* = 21)	40 Females 18–40 years	Cross-sectional, observational 16S rRNA gene amplification of the V3 and V4 regions	Acceleration, energy expenditure, intensity of physical activity and body position measured by Acti -Sleep V.3.4.2 accelerometer. Dietary pattern assessed by 97 food item FFQ.	• Physical activity associated with increased the abundance of health-promoting bacteria (*Bifidobacterium* species*, Roseburia hominis, A. muciniphila* and *Faecalibacterium prausnitzii*) in the microbiota. • Inverse association between sedentary parameters and microbiota richness (number of species, and Shannon and Simpson indices).
3. Mörkl et al., 2017, Austria [57]	Anorexia nervosa patients (*n* = 18), athletes (*n* = 20), normal weight (*n* = 26), overweight (*n* = 22), and obese women (*n* = 20).	106 Females 18–40 years	Cross-sectional, observational 16S rRNA gene amplification of the V1 and V2 regions	24-h recall dietary intake Activity level assessed by IPAQ^f^ score & MET^g^/min	• Lower microbial richness in obese and anorexic individuals compared to athletes.
4. Petersen et al., 2017, USA [18]	Professional (*n* = 22) and amateur (*n* = 11) level competitive cyclists	22 Males/ 11 Females 19–49 years	Cross-sectional, observational Metagenomic whole genome shotgun sequencing and RNA sequencing	Exercise load (hours/week): 6–10; 11–15; 16–20; and; 20 + . Diet: equal protein, fat, carbohydrate; vegetarian; high complex carbohydrate; paleo; gluten-free.	• No significant correlations between taxonomic cluster and professional or amateur level cyclists. • High relative abundance of *Prevotella* in cyclists training > 11 h/week. • Increased abundance of *Methanobrevibacter smithii* transcripts in professional cyclists.
5. Barton et al., 2018, Ireland [13]	Professional male rugby athletes (*n* = 40) and healthy size, age and gender matched controls (*n* = 46)	86 Males 23–35 years	Cross-sectional, observational Metagenomic whole genome shotgun sequencing and urine and fecal metabolomics	Diet recorded by a 187-food item FFQ. Physical activity levels assessed by EPIC-Norfolk questionnaire. Serum creatine kinase levels were used as a proxy for level of physical activity.	• The microbiota of athletes was more diverse than both the low and high BMI control groups at the functional level. • Athletes had an enriched profile of SCFAs^h^ and higher levels of the metabolite TMAO^i^.
6. Jang et al., 2019, South Korea [11]	Healthy sedentary men (as controls; *n* = 15), bodybuilders (*n* = 15), and elite distance runners (*n* = 15)	45 Males 19–28 years	Cross-sectional, observational 16S rRNA gene amplification of the V3 and V4 regions	Physical activity level was assessed using the IPAQ. Dietary intake was analyzed with the computerized nutritional evaluation program.	• Exercise type was associated with athlete diet patterns (bodybuilders: high-protein, high-fat, low-carbohydrate, and low-dietary fiber diet; distance runners: low-carbohydrate and low dietary fiber diet). Athlete type was significantly associated with the relative abundance of gut microbiota at the genus and species level. • Increased abundance of *Faecalibacterium*, *Sutterella*, *Clostridium*, *Haemophilus*, and *Eisenbergiella* in bodybuilders. • Athlete type did not differ in gut microbiota alpha and beta diversity.
7. O’Donovan et al., 2019, Ireland [58]	Elite athletes from 16 different sports (*n* = 37)	23 Males/ 14 Females 27 ± 5 years	Cross-sectional, observational Metagenomic whole genome shotgun sequencing and urine and fecal metabolomics	Diet recorded by FFQ.	• Microbial diversity did not differ between sport classification. • Overall, samples dominated by species from one or a combination of five species: *Eubacterium rectale*, *Polynucleobacter necessarius*, *Faecalibacterium prausnitzii*, *Bacteroides vulgatus* and *Gordonibacter massiliensis.* • Athletes with high dynamic^j^ component were associated with greater abundance of *Bifidobacterium animalis*, *Lactobacillus acidophilus*, *Prevotella intermedia* and *F. prausnitzii*. • Athletes with both a high dynamic and static^k^ component were associated with greater abundance of *Bacteroides caccae.*

^a^*FFQ* Food frequency questionnaire

^*b*^*EPIC* European Prospective Investigation of Cancer

^c^*GM* Gut microbiota

^d^*BMI* Body mass index

^e^*WHO* World Health Organization

^f^*IPAQ* International Physical Activity Questionnaire

^g^*MET* Metabolic equivalent

^h^*SCFA* Short-chain fatty acid

^i^*TMAO* Trimethylamine N-oxide

^j^*Dynamic* Classified by estimated VO_2_max

^k^*Static* Classified by maximal voluntary contraction

#### Athletes/physically active individuals vs other populations

Several studies have investigated the difference in compositional gut microbiota between those physically active (including athletes) and a range of populations. While the above confounding factors are still relevant when interpreting these studies, such research provides an important comparator to the gut microbiota of these individuals. In an observational study, the gut microbiotas of sedentary and physically active premenopausal women were compared [56]. Physical exercise was not related to differences in the microbiota diversity or richness (the total number of OTUs or species recorded); however, sedentary parameters (i.e., sedentary time and breaks) correlated negatively with microbiota richness. Further, quantitative polymerase chain reaction analysis revealed higher abundance of health-promoting bacterial species in active women, including *Faecalibacterium prausnitzii*, *Roseburia hominis* and *Akkermansia muciniphila*. In another cross-sectional study in females, Mörkl et al. [57] compared the gut microbiota of anorexia nervosa inpatients to recreational athletes from a range of sports and overweight, obese, and normal weight controls. Microbiota diversity was markedly lower in anorexia nervosa patients and obese participants compared to other groups, while athletes showed the highest alpha diversity (species richness). Interestingly, total fat mass, serum lipids, C-reactive protein, depression scales, and smoking status were negatively associated (*R*^2^ = − 0.012 to − 0.256, *P* < 0.05) with microbiota diversity. It is important to note that these associations are likely driven by lifestyle. While caution should be used in interpreting this data given the cross-sectional nature and inability to account for other potential influences, investigation of gut microbiota composition, diversity, and function should be useful in characterizing key elements of a healthy lifestyle.

Clarke et al. [19] reported the gut microbiota of professional male rugby players was more diverse than healthy, non-athlete subjects matched for body mass index (BMI), age, and gender. Given the physical size of modern rugby players, two control groups were assessed; one matched for athlete size with a comparable (elevated) BMI (> 28 kg/m^2^) and a second reflecting the background age-matched and sex-matched population (lower BMI of < 25 kg/m^2^). Importantly, the alpha diversity of the elite athlete’s microbiota was higher than that of both control groups. Further, the athletes and those in the low BMI control group had higher proportions of the genus *Akkermansia* than the high BMI control group. Moreover, protein consumption was correlated positively (*R* = 0.24–0.43) with microbial diversity across all groups, indicating that greater protein intake was linked to higher levels of microbial diversity. There is a possibility that the increased diversity of the athlete’s gut microbiota was due, in part, to their higher protein intake. Barton et al. [13] re-examined the microbiota in these participants using whole metagenome shotgun sequencing to provide deeper insight into taxonomic composition and metabolic potential. Differences in fecal microbiota between athletes and sedentary controls showed even greater separation at the metagenomic and metabolomic level than at the gut microbiota compositional level. Relative to controls athletes appear to have increases in metabolic pathways (e.g., amino acid and antibiotic biosynthesis and carbohydrate metabolism) and fecal metabolites (e.g., microbial produced short-chain fatty acids (SCFAs) including acetate, propionate, and butyrate) associated with enhanced fitness and overall health when compared to control groups [13].

#### Athletes across different classifications and disciplines

To explore possible differences between levels of athletes, Petersen et al. [18] investigated the gut microbiotas of 22 professional and 11 amateur competitive cyclists. Using metagenomic whole genome shotgun sequencing, no significant correlations were evident between the taxonomic clusters in professional or amateur cycling status. However, the amount of exercise (time reported exercising during the average week) was correlated positively with a greater abundance of the genus *Prevotella* (≥2.5%). Increased abundance of *Prevotella*, common in non-Western populations and associated with plant/fiber rich diets, was further positively correlated with several amino acid and carbohydrate metabolism pathways, including branched-chain amino acid (BCAA) metabolism. Using metatranscriptomic sequencing, there was increased abundance of *Methanobrevibacter smithii* transcripts in a number of the professional cyclists in comparison to amateur cyclists. Moreover, this archaeon had upregulation of genes involved in the production of methane and when methane metabolism was upregulated, there was similar upregulation of energy and carbohydrate metabolism pathways. Similar to Clarke et al. [19], there was low abundance of *Bacteroides* in the athletes. In addition, *Akkermansia* was present in 30 out of 33 cyclists, with seven cyclists having relative abundances > 2% of this microbe in their metagenomic community. These outcomes may have been a reflection of higher fitness/metabolism of these athletes as increased proportions of *Akkermansia* are generally associated with a healthier metabolic profile [59].

To examine the gut microbiota and metabolome across sport classification, O’Donovan and colleagues [58] collected fecal and urine samples from 37 elite Irish athletes across 16 different sports (many of whom were participating in the 2016 Summer Olympics). To gain an understanding of the impact of dynamic (classified by estimated VO_2_max) versus static (classified by maximal voluntary contraction) components of exercise, each sport was classified into a broader sports classification group. Fecal samples were prepared for shotgun metagenomic sequencing and fecal and urine samples underwent metabolomic profiling. Athletes participating in sports with a high dynamic component were the most distinct compositionally (greater differences in proportions of species; see Table 1), while athletes participating in sports with both a high dynamic and static component were the most functionally distinct (greater differences in functional potential). Fecal and urine derived metabolites also varied between classification including increased lactate (urine) in sports with a static component and creatinine (feces) in sports with a high dynamic and low static component. While increased lactate in the more static-based sports was not surprising, increased creatinine in more dynamic-based sports was. It may have been that the dynamic exercises in this athletic cohort involved substantial muscle turnover, potentially resulting in the increase in the production of creatinine. It is unclear what the causative factors of this finding and other reported differences in the gut microbiota and metabolome were due to the sampling variation, sample size and cross-sectional nature of this study. However, these differences were observed despite no significant variation in dietary intakes across athletes from different classifications; suggesting that variations in training loads and competition requirements contributed to these microbiome and metabolome-related patterns.

To investigate the long-term effects of a specific exercise type and athletes’ diets on gut microbiota, Jang et al. [11] compared fecal microbiota characteristics, dietary intake, and body composition in 15 healthy sedentary men (as controls), 15 bodybuilders, and 15 distance runners. Exercise type was associated with athlete diet patterns (i.e., bodybuilders: high-protein, high-fat, and low-carbohydrate/dietary fiber diet; distance runners: low-carbohydrate and low-dietary fiber diet). While athlete type did not differ regarding gut microbiota alpha and beta diversity, it was significantly associated with the relative abundance of several bacteria. For instance, at the genus level, *Faecalibacterium*, *Sutterella*, *Clostridium*, *Haemophilus*, and *Eisenbergiella* were the highest, while *Bifidobacterium* and *Parasutterella* were the lowest in bodybuilders. At the species level, intestinal beneficial bacteria widely used as probiotics (*Bifidobacterium adolescentis*, *Bifidobacterium longum*, *Lactobacillus sakei*) and those producing short chain fatty acids (*Blautia wexlerae*, *Eubacterium hallii*) were the lowest in bodybuilders and the highest in controls. In distance runners, protein intake was negatively correlated with diversity (Shannon index *R* = − 0.63; *P* = 0.01) and in bodybuilders, fat intake was negatively correlated with *Bifidobacteria* (*R* = − 0.52; *P* = 0.05). These differences may relate to the nutrition status of athletes in the study (i.e. insufficient carbohydrate and dietary fiber; higher fat).

#### Summary of the athlete/exercise-associated gut microbiota

From the limited evidence, it appears that athletes harbor an increased abundance of functional pathways within the microbiome that are exploited by the host for potential health benefits, as well as carbohydrate degradation and secondary metabolite metabolism compared to control groups [60]. While difficult to isolate, the influence of dietary intake on many of these differences is likely and needs to be considered in the context of this research. Furthermore, athletes have an enriched profile of SCFAs, previously associated with numerous health benefits and a lean phenotype [61–63]. This profile along with an increased number of detected SCFA pathways in athletes is conducive to an enhanced rate of SCFA production [64]. While evidence is currently limited, results from Clarke et al. [19] and Petersen et al. [18] suggest *A. muciniphilia* may be more present in athletes than non-athletes. *A. muciniphilia* is a mucin-degrading bacterium that resides in the nutrient-rich mucus layer of the gut that appears to be associated with positive metabolic function [59]. *A. muciniphilia* levels are decreased in obese and type 2 diabetic mice, and treatment with these bacteria reversed high-fat diet-induced metabolic disorders, including fat mass gain, metabolic endotoxemia, adipose tissue inflammation, and insulin resistance [65]. *A. muciniphilia* can control mucus production by the host and restore mucus layer thickness in mice with high-fat diet-induced obesity, thereby reducing gut permeability. This outcome led to the hypothesis that *A. muciniphilia* engages in cross-talk with the intestinal epithelium to control inflammation and gut barrier function [65]. *A. muciniphilia* has also been found to be enriched in mice fed a ketogenic diet, exhibiting gut-brain functions including conferring seizure protection in two preclinical models for refractory epilepsy [66]. While the role of *A. muciniphila* is less certain in humans, it is depleted in individuals with several metabolic and inflammatory disorders. For example, of subjects undergoing dietary calorie restriction treatment for obesity, those with higher levels of these bacteria exhibited the best metabolic status and clinical outcomes [59]. Future research in athletes should continue to investigate the role *A. muciniphilia* plays in the gut microbiota and its functional impact on metabolism.

In relation to obesity, some athletes who may be defined as physically active may not necessarily be healthier based on BMI [67]. For example, Rugby or American football players commonly have large amounts of lean mass, and many will have relatively healthy percent body fat levels, typically 12–20%, so BMI is considered to be a poor measure of obesity status in these athletic cohorts. While Clarke et al. [19] and Barton et al. [13] compared the gut microbiome of athletes to matched controls considered overweight by BMI, future work should also investigate this comparison at the obese classification. Findings from such research could provide important data in connection with the pathogenesis of obesity and the gut microbiota. One leading theory on the pathogenesis of obesity emphasizes a close link between the metabolic and immune systems via the gut microbiota [68]. This body of work suggests that increased intestinal permeability from high-fat / high sugar diets allows bacterial lipopolysaccharide (LPS), an outer membrane component of gram-negative bacteria linked with induction of inflammation, from the gut microbiota to translocate into the systemic circulation, resulting in systemic endotoxemia. Activation of pro-inflammatory cytokines is observed, leading to the chronic low-grade inflammation often implicated in obesity [69]. In contrast, athletes have been noted to have lower levels of circulating LPS compared to sedentary individuals [70]. These findings support of the notion that other factors beyond BMI levels should be considered when assessing any relationship between metabolic health, obesity and the microbiome status of competitive athletes.

Barton et al. [13] speculated that the athlete gut microbiome may possess a functional capacity primed for tissue repair and a greater ability to harness energy from the diet with increased capacity for carbohydrate metabolism, cell structure, and nucleotide biosynthesis. This assertion reflects the significant energy demands and tissue adaptation that occurs during intense exercise and elite sport. It appears that being physically active is another important factor in the relationship between the microbiota and host metabolism. Intervention-based studies to delineate this relationship will be important and provide further insights into optimal therapies to influence the gut microbiota, and its relationship with health and disease as well as athletic performance.

**Table:** 

**Key Points 1 – Athlete/exercise-associated microbiota**	
• Although limited in evidence, active individual’s microbiota display a higher abundance of health-promoting bacterial species such as *A. muciniphila* and increased diversity.	
• Body composition and physical activity are positively correlated with several bacterial populations.	
• Investigating metagenomic functions rather than taxonomy alone provides a more meaningful understanding of gut microbiota and the impact of microbial metabolic functions on human physiology.	
• Athletes have more fecal metabolites (e.g., microbial SCFAs including acetate, propionate, and butyrate) associated with enhanced muscle turnover (fitness) and overall health than less active individuals. These differences are likely driven by the effects of exercise training and/or dietary intake.	
• The athlete/exercise-associated gut microbiome may possess a functional capacity primed for tissue repair and a greater ability to harness energy from the diet with increased capacity for carbohydrate metabolism, cell structure, and nucleotide biosynthesis.	

### The effect of exercise on the gut microbiota

#### Animal research

Few studies have focused on the impact that voluntary exercise has on gut microbiota and, to date, all but seven of these experimental studies utilized murine models [17, 71]. These preliminary studies indicate that exercise influences the composition of the gut microbiota community. Matsumoto et al. [72] reported that regular running exercise in rats was related to an increase in butyrate-producing bacteria in the microbiota composition along with an increase of butyrate concentration. Other animal studies demonstrated that daily wheel running exercise may improve some aspects of unhealthy states, such as diet-induced obesity, diabetes, and toxicity, by impacting the gut microbial composition in mice [73–75]. These effects included altering the ratio between the dominant phyla *Firmicutes* and *Bacteroidetes*, which has been found to be correlated with obesity and other diseases [76–78]. Animal and human data have reported that the *Firmicutes/Bacteroidetes* ratio is higher (i.e., increased *Firmicutes* and/or decreased *Bacteroidetes*) in obese people compared to lean people [79–82]. However, this is not always the same between studies [83, 84] and may be an oversimplification of phylum-level patterns in relation to host health. Further, mechanisms by which specific members of the microbiota, such as *Firmicutes* and *Bacteroidetes*, can affect human phenotypes remain to be fully elucidated [85]. Therefore, changes in phyla ratios should be interpreted with caution.

Amongst the exercise studies in animals there was little agreement regarding what taxa are influenced by chronic exercise. Other than a positive correlation between exercise and *Lactobacillus* [73, 86, 87], there are no other taxa that consistently increase in relative abundance in regularly exercised mice or rats. In particular, Choi et al. [73] reported that, in comparison to a sedentary control, mice with running wheel exercise had higher phylum *Firmicutes* but fewer phyla *Tenericutes* and *Bacteroidetes*, which attenuated changes in gut microbiota induced by oral exposure to the environmental toxin, polychlorinated biphenyls. Lambert et al. [75] described that exercised mice presented a greater abundance of *Firmicutes* species and lower *Bacteroides/Prevotella* genera compared with sedentary mice. In contrast, Evans et al. [74] concluded that exercise increased *Bacteroidetes*, while it decreased *Firmicutes* in mice, implying that exercise plays an important role in prevention of diet-induced obesity producing a microbial composition similar to lean mice. The changes in the *Firmicutes/Bacteroidetes* ratio exerted by exercise were inversely proportional to the distance traveled by animals [74]. Campbell et al. [88] asserted that exercised mice have bacteria related to *Faecalibacterium prausnitzii* which may protect the digestive tract by producing butyrate and lowering the oxygen tension in the lumen by a flavin/thiol electron shuttle. Briefly, the health-related effects of butyrate are associated with anti-inflammatory properties, direct feeding of colonocytes, and an impact on satiety [64]. Notably, butyrate, along with propionate and acetate, provides ~ 10% of the daily caloric requirements in humans who consume high (~ 60 g/day) amounts of dietary fiber [64]. The bacterial abundance of *Clostridiaceae* and *Bacteroidaceae* families and *Ruminoccocus* genus were negatively associated with blood lactate levels in exercised animals, whereas a positive association was reported for *Oscillospira* genus [86].

Moreover, it seems that the changes exerted by physical exercise depend on the physiological state of the individual. For example, regular forced exercise differentially affected microbiota richness whether they were obese-hypertensive or normal rats [86]. Alterations to the microbiota exerted by exercise in rats following a high-fat diet were different to rats on a normal diet [89], as well as the alterations produced in diabetic mice were different to their control counterparts [75]. Collectively, these findings indicate that modulation of the microbiota by chronic exercise depends not only on the physiological state of the individual, but also on the diet. Moreover, another significant difference between forced vs. voluntary exercise in animals is exercise volume. This is recapitulated in human cyclist data [18] and requires further investigation in animal models. Finally, it has been observed that exercise induces more effective changes in the microbiota in juvenile rats than in adult rats [90]. A common finding in these murine studies examining the effects of exercise training on the gut microbiome, is an increase in alpha diversity [17]. Several other studies using murine-based models also demonstrated increased alpha diversity in animals that exercised vs. those that were sedentary [73, 74, 86, 87].

#### Cross-sectional research in humans

In healthy individuals, Estaki et al. [14] reported higher cardiorespiratory fitness (as measured by peak or maximum oxygen uptake, VO_2_peak or VO_2_max) correlated with increases in both microbial diversity and fecal butyrate (see Table 2). Also identified was a core set of gene related functions rather than a core set of bacterial taxa in individuals with high levels of fitness [14]. Further, ~ 20% of the variation in gut bacterial alpha diversity could be explained by VO_2_peak alone; in fact, VO_2_peak stood as the only variable that contributed significantly to increased alpha diversity. The primary findings indicate that cardiorespiratory fitness is a good predictor of gut microbial diversity in healthy humans, outperforming several other variables including sex, age, BMI, and multiple dietary components. Additionally, enhanced bacterial diversity was correlated positively with certain microbial metabolic functions including chemotaxis, motility, and fatty acid biosynthesis. As VO_2_peak was not significantly associated with variation in community composition, this result suggests function may be a better predictor than species richness, as noted previously. This study also confirmed results by Matsumoto and colleagues [72] who initially reported that exercising rats exhibited a positive correlation between high-cardiorespiratory fitness and an increase in the SCFA, butyrate. Increases in fecal butyrate were observed when relative abundances of *Clostridiales*, *Roseburia*, *Lachnospiraceae*, and *Erysipelotrichaceae* were increased [14]. These SCFAs are derived from fermentation of undigested plant fiber by the microbiota in the large intestine.

**Table 2:** Effect of exercise and/or athletic diet on the gut microbiota: Characteristics of included articles (by publication date)

Authors, year, country	Subjects characteristics	Study design and gut microbiome analysis	Diet and/or exercise	Duration	Key outcome(s)
1. Clarke et al., 2014, Ireland [19]	Professional male rugby players, *n* = 40, 29 ± 4 years; Healthy matched controls, *n* = 46, 29 ± 6 years	Cross-sectional 16S rRNA gene amplification of the V4 region	Observational	Cross-sectional	• Athletes had a higher diversity of gut micro-organisms, representing 22 distinct phyla. • GM^a^ diversity indices were positively correlated with protein intake and serum creatine kinase in athletes.
2. Estaki et al., 2016, Canada [14]	Healthy young males and females, *n* = 39, stratified by cardiorespiratory fitness, Low: 25.5 ± 3.3 years; Average: 24.3 ± 3.7 years; High: 26.2 ± 5.5 years	Cross-sectional 16S rRNA gene amplification of the V3 and V4 regions	Observational	Cross-sectional	• VO_2_peak^b^, independent of diet, positively correlated with increased GM diversity. • VO_2_peak explained significant variation in GM predicted metagenomic functions, aligning positively with genes related to bacterial chemotaxis, motility, and fatty acid biosynthesis. • Increased production of fecal butyrate abundances of butyrate-producing taxa (*Clostridiales*, *Roseburia*, *Lachnospiraceae*, and *Erysipelotrichaceae*) amongst physically fit participants.
3. Yang et al., 2017, Finland [94]	Premenopausal females with low, moderate and high-cardiorespiratory fitness (VO_2_max), *n* = 71, Low VO_2_max: 40.4 ± 3.5 years; Moderate VO_2_max: 39.7 ± 4.2 years; High VO_2_max: 30.6 ± 5 years	Cross-sectional 16S rRNA hybridization, DNA-staining, and flow cytometry	Observational	Cross-sectional	• Decreased *Bacteroides*, increased *Eubacterium rectale-Clostridium coccoides* in low VO_2_max participants vs high VO_2_max participants. • VO_2_max inversely associated with *Eubacterium rectale-C. coccoides* but not with other bacteria.
4. Allen et al., 2018, USA [98]	Sedentary lean males and females, *n* = 18, 25.1 ± 6.52 years; Obese males and females, *n* = 14, 31.14 ± 8.57 years	Longitudinal design 16S rRNA gene amplification of the V4 region	30–60 min moderate-to-vigorous intensity (60–75% of HRR^c^) aerobic exercise, 3x per week	6 weeks	• Exercise induced shifts in SCFA^d^-producing taxa (*Faecalibacterium* and *Lachnospira* species) and genetic machinery (BCoAT^e^) were more substantial in lean versus obese participants. • A return to sedentary activity for 6 weeks led to a BMI^f^-dependent reversion in gut microbiome composition.
5. Barton et al., 2018, Ireland [13]	Professional male rugby players, *n* = 40, 29 ± 4 years; Healthy matched controls, *n* = 46, 29 ± 6 years	Cross-sectional Metagenomic whole genome shotgun sequencing and urine and fecal metabolomics	Observational	Cross-sectional	• Relative increase in pathways (amino acid and antibiotic biosynthesis and carbohydrate metabolism) in athletes vs control. • Increase in fecal metabolites (acetate, propionate and butyrate) in athletes vs control.
6. Cronin et al., 2018, Ireland [199]	Sedentary overweight/obese males and females, *n* = 90, 18–40 years	Randomized controlled trial, parallel group design Metagenomic whole genome shotgun sequencing and urine and fecal metabolomics	Groups: • Protein-only; 30 g protein (24 g whey) • Exercise-only; Combined aerobic and resistance training (aerobic training of moderate intensity and resistance training of 3 sets of 8 repetitions on 7 different resistance machines) 3 x per week • Exercise + protein	8 weeks	• Increase in alpha diversity in Exercise + Protein group vs Protein group. • Decrease in beta diversity of the gut virome in participants consuming protein.
7. Moreno-Pérez et al., 2018, Spain [196]	Endurance-trained males, *n* = 18, 35.38 ± 9.0 years (control group), 34.90 ± 9.49 years (protein group)	Randomized controlled trial, parallel group design 16S rRNA gene amplification of the V3 and V4 regions	Groups: • Control: maltodextrin • Protein: blend of whey isolate (10 g) and beef hydrolysate (10 g) Minimum training frequency of 5 endurance training sessions per week; ≥240 min per week	10 weeks	• Increase in *Bacteroidetes* in protein group. • Decrease in *Roseburia*, *Blautia*, and *Bifidobacterium longum* in protein group.
8. Taniguchi et al., 2018, Japan [101]	Healthy elderly Japanese males, *n* = 33, 62–76 years	Randomized crossover trial 16S rRNA gene amplification of the V3 and V4 regions	5-week control period OR 5-week supervised, progressive aerobic exercise program. Cycle ergometer 3x per week. 60% of pre exercise VO_2_peak during week 1, 70% during weeks 2 and 3, and 75% during weeks 4 and 5. Duration was 30 min for weeks 1 and 2, and 45 min for weeks 3–5.	5 weeks	• Short-term endurance exercise did not appreciably influence diversity and composition of gut microbiota. Minor changes in the gut microbiota were associated with cardiometabolic risk factors. • Decreased relative abundance of *C. difficile* significantly decreased, whereas *Oscillospira* significantly increased during exercise as compared to the control period.
9. Durk et al., 2019, USA [93]	Healthy young males and females, *n* = 37, 25.7 ± 2.2 years	Cross-sectional Quantitative Polymerase Chain Reaction (qPCR) that specifically measured the quantity of a target gene (16 s RNA) found in *Firmicutes* and *Bacteroidetes*	Observational	Cross-sectional	• *Firmicutes/Bacteroidetes* ratio positively correlated to VO_2_max. • VO_2_max explained ~ 22% of the variance of an individual’s relative gut bacteria as determined by *Firmicutes/Bacteroidetes* ratio.
10. Keohane et al., 2019, Ireland [99]	Ultra-endurance male athletes, *n* = 4, 26.5 ± 1.3 years	A prospective, repeated-measures, within-subject report Metagenomic whole genome shotgun sequencing	Observational	33-day event; 3-month follow-up	• Increased alpha diversity throughout event. • Increased abundance of butyrate producing species (*Roseburia hominis* and members of the genus *Subdoligranulum*) and species associated with improved metabolic health (*Dorea longicatena*). • Many of the adaptions in GM community structure and metaproteomics persisted at 3 months follow up.
11. Kern et al., 2019, Denmark [108]	Overweight/obese males and females; *n* = 88; 20–45 years	Randomized controlled trial, parallel group design 16S rRNA gene amplification of the V4 region	Exercise groups: • Habitual living (CON), • Active commuting by bike (BIKE) • Leisure-time exercise of moderate intensity (MOD) • Vigorous intensity exercise (VIG)	6 months	• Increase alpha diversity index in VIG at 3 months compared with CON. • Beta diversity changed in all exercise groups compared with CON; VIG decreased heterogeneity. • Increased inferred functional potential of microbiota in the exercise groups, primarily at 3 months and in MOD.
12. Morita et al., 2019, Japan [103]	Healthy sedentary elderly females; *n* = 32; ≥ 65 years	Non-randomized comparative trial 16S rRNA terminal restriction fragment length polymorphism analyses	Exercise groups: • Trunk muscle training group 1 h per week. • Aerobic exercise training group, brisk walking at ≥3 METS^g^, 1 h daily.	12 weeks	• Increased *Bacteroides* relative abundance in aerobic exercise group. • Increased *Bacteroides* following exercise intervention associated with increased 6-min walk test.
13. Motiani et al., 2019, Finland [109]	Obese sedentary, prediabetic/type 2 diabetic males and females; *n* = 26 (*n* = 9, *n* = 17 respectfully); 49 ± 4 years	Parallel group design 16S rRNA gene amplification of the V3 and V4 regions	Exercise groups: • Sprint interval training group; 30 s exercise bouts (4–6) of all out cycling efforts with 4 min of recovery, 3x a week. • Moderate intensity continuous training group; 40–60 min cycling at 60% of VO_2_peak intensity, 3x a week.	2 weeks	• Increased *Bacteroidetes* in both groups. • Decreased *Firmicutes:Bacteroidetes* ratio in both groups. • Decreased *Clostridium* genus and *Blautia*. • Colonic glucose uptake positively associated with *Bacteroidetes* and inversely with *Firmicutes* phylum, *Firmicutes: Bacteroidetes* ratio and *Blautia* genus.
14. Murtaza et al., 2019, Australia [230]	Elite male endurance race walkers; *n* = 21; 20–35 years	Non-randomized comparative trial 16S rRNA gene amplification of the V6-V8 regions	Diet groups: • High-Carbohydrate • Periodized Carbohydrate • ketogenic LCHF^h^ *Consumed during an intensified training program.*	3 weeks	• Microbiota profiles at baseline could be separated by *Prevotella* or *Bacteroides* dominated enterotype. • LCHF diet resulted in increased relative abundance of *Bacteroides* and *Dorea* and decreased *Faecalibacterium*. • Negative correlations between *Bacteroides* and fat oxidation, and between *Dorea* and economy test following LCHF intervention.
15. Scheiman et al., 2019, USA [16]	Experiment 1: • Athletes from the 2015 Boston Marathon (*n* = 15), sedentary controls (*n* = 10) Experiment 2: • Ultramarathoners and Olympic trial rowers (*n* = 87)	Observational 16S rRNA gene amplification of the V4 region for experiment 1 Metagenomic whole genome shotgun sequencing for experiment 2	Experiment 1: • Marathon event Experiment 2: • Exercise bout	Experiment 1: • Fecal samples collected 7 days before and after Marathon event Experiment 2: • Pre/post exercise	Experiment 1: • Increased *Veillonella* relative abundance in marathon runners post marathon. • *Veillonella* was more prevalent among runner’s vs non-runners, although this was not statistically significant. Experiment 2: • Increased *Veillonella* abundance post exercise. • *Veillonella* methylmalonyl-CoA pathway was overrepresented in athlete metagenomic samples post exercise.
16. Liu et al., 2019, China [106]	Overweight/obese prediabetic males; *n* = 39; 20–60 years	Randomized controlled trial, parallel group design Metagenomic whole genome shotgun sequencing and fecal metabolomics	Groups: • Control; No exercise. • High-intensity exercise; 70 min combined aerobic and resistance interval training, 3x a week.	12 weeks	• Microbiota profiles were differentially altered in exercise responders (*n* = 14) vs non-responders (*n* = 6). • The microbiome of responders had increased functional capacity for SCFA biosynthesis and BCAA^i^ catabolism. • Exercise-induced gut microbiota changes were positively correlated with improvements in glucose homeostasis and insulin sensitivity. • Baseline microbiome features accurately predicted personalized exercise responses. • Fecal microbiota transplantation from responders conferred the metabolic benefits of exercise in mice.

^a^*GM* Gut microbiota

^b^*VO*_*2*_ Volume of oxygen utilization

^c^*HRR* Heart rate reserve

^d^*SCFA* Short-chain fatty acid

^e^*BCoAT* Butyryl-CoA:acetate CoA-transferase, a butyrate-regulating gene

^f^*BMI* Body mass index

^g^*METS* Metabolic equivalents

^h^*LCHF* Low-Carbohydrate High-Fat

^i^*BCAA* Branched-chain amino acid

Functional categories most strongly correlated with VO_2_peak were related to bacterial motility (bacterial motility proteins, flagella assembly, and bacterial chemotaxis), sporulation, and to a lesser extent the two-component system known to enable bacterial communities to sense and respond to environmental factors [14]. One possible mechanism behind these associations may be derived from the observation that butyrate, which was more abundant among participants with higher cardiorespiratory fitness, can modulate neutrophil chemotaxis [91]. VO_2_peak was inversely correlated with LPS biosynthesis and LPS biosynthesis proteins which were elevated among less fit participants. LPS is a major component of the cell wall of gram-negative bacteria and considered an endotoxin when present in the blood. By binding to extracellular toll-like receptor 4 located on many cell types, LPS elicits strong inflammatory responses that may be detrimental to the host. Continuous low-level translocation of LPS into circulation can induce chronic low-level inflammatory states associated with development of obesity and other metabolic syndromes [92]. These inflammatory states are thought to be derived to some extent from inflammatory responses to blood LPS which is elevated in sedentary humans [70]. Exercise training attenuates inflammation in part by reducing elevated blood LPS [70]. The inverse relationship between VO_2_peak and LPS biosynthesis pathways implies a beneficial consequence of increased physical activity which may result in decreased LPS biosynthesis.

Durk et al. [93] also explored the relationship between cardiorespiratory fitness and relative gut microbiota composition in healthy young adults showing that *Firmicutes/Bacteroidetes* ratio was significantly positively correlated to VO_2_max. While no other relationships between the gut microbiota and fitness, nutritional intake, or anthropometric variables were found, VO_2_max accounted for ~ 22% of the variance of an individual’s relative gut bacteria (as determined by the *Firmicutes/Bacteroidetes* ratio). In a cross-sectional study in premenopausal women, cardiorespiratory fitness was associated with gut microbiota composition, independent of age and carbohydrate or fat intake [94]. Participants with low VO_2_max had lower *Bacteroides*, but higher *Eubacterium rectale-Clostridium coccoides* than the high VO_2_max group. Aerobic capacity was inversely associated with *Eubacterium rectale*-*Clostridium coccoides* but not with other bacteria. After adjusting for age and dietary intake, all significant associations remained.

In professional rugby players, who had a unique dietary pattern (higher energy intake and quantities of protein, fat, carbohydrates, sugar and saturated fat per day, with protein accounting for considerably more of the total energy) and a high level of physical activity, there was a higher diversity of gut microbiota compared with controls [19]. However, it was unclear if this effect was due to exercise, a high-protein diet, a combination of these two factors, or other factors [19].

#### Acute exercise effects on the human gut microbiota

To investigate the effect of an acute exercise bout in athletes, Zhao et al. [20] examined the fecal metabolites and microbiota in 20 amateur runners before and after a half-marathon race using metabolomics and 16S rRNA sequencing analysis. According to the alpha diversity analysis, there were few differences in diversity, nevertheless, abundances of certain microbiota members showed differences before and after running. At the phylum level, *Lentisphaerae* and *Acidobacteria*, whose functions in the human gut are unknown, were detected after running. At the species level there was a marked increase in the families *Coriobacteriaceae* and *Succinivibrionaceae*. *Coriobacteriaceae* (phylum *Actinobacteria*) is involved in metabolism of bile salts and steroid hormones as well as activation of dietary polyphenols in the human gut [95]. *Coriobacteriaceae* was correlated positively with 15 metabolites, indicating that metabolism of *Coriobacteriaceae* may be a potential mechanism underlying the role of exercise in preventing disease and improving health outcomes. These increased metabolites indicate a microbiota-derived metabolism was promoted by running. At the genus level, half-marathon running reduced the abundance of fecal *Ezakiella*, *Romboutsia,* and *Actinobacillus,* but increased the abundance of *Coprococcus* and *Ruminococcus bicirculans*. *Actinobacillus* species are purportedly responsible for several distinct animal diseases, such as actinomycosis in cattle, potent septicemia in the neonatal foal, and human periodontal disease [96]. Thus, inhibition of this potential pathogen indicated an anti-inflammatory effect of exercise. Interestingly, the pentose phosphate pathway, a metabolic pathway parallel to glycolysis and involving the oxidation of glucose, was the most enriched pathway after a half-marathon run. These findings highlight a microbiota-derived mechanism for the health-promoting benefits of exercise.

In a unique study by Scheiman et al. [16], athletes who were to run the Boston Marathon were recruited, along with a set of sedentary controls to identify gut bacteria associated with athletic performance and recovery states. 16S ribosomal DNA sequencing was conducted on daily fecal samples collected up to 1 week before and 1 week after the marathon event. The relative abundance of the bacterial genus *Veillonella* increased after the marathon and was the most differentially abundant microbial feature between the pre- and post-exercise states. Additionally, *Veillonella* was more prevalent among runners compared to non-runners. *Veillonella* species metabolize lactate into the SCFA acetate and propionate via the methylmalonyl-CoA pathway [97]. To replicate these results in a second experiment and an additional cohort of human athletes, Scheiman and colleagues [16] performed shotgun metagenomic sequencing on stool samples from ultramarathoners and Olympic trial rowers both before and after an exercise bout. Similar findings were reproduced as relative taxonomic abundances *Veillonella* were increased post-exercise. In addition, the *Veillonella* methylmalonyl-CoA pathway was overrepresented in the metagenomic samples post-exercise across the cohort. Given the limited prevalence of the methylmalonyl-CoA pathway across lactate-utilizing microbes, this enrichment post-exercise may implicate *Veillonella* in causing functional changes in the metabolic repertoire of the gut microbiota. It seems that the genus *Veillonella* is enriched in athletes after exercise and the metabolic pathway that *Veillonella* species utilize for lactate metabolism is also enriched. Gut colonization of *Veillonella* may augment the Cori cycle by providing an alternative lactate-processing method whereby systemic lactate is converted into SCFAs that re-enter the circulation. Higher levels of lactic acid in athletes’ GI tract favor the growth of this genus and these bacteria may in turn produce a compound that could aid performance.

In a third set of experiments, Scheiman et al. [16] isolated a strain of *Veillonella atypica* from a stool sample of one of the aforementioned marathon runners and inoculated mice. In a pre-clinical crossover trial, *Veillonella* inoculated mice had a 13% improvement in time to exhaustion running tests as well as significant reductions in inflammatory cytokines post exercise compared to control. They also more effectively converted lactate to the SCFAs propionate. Importantly, Scheiman et al. [16] found systemic lactate in these animals was able to cross the gut barrier into the lumen, making it available as a substrate for microbial SCFA conversion. Taken together, these experiments revealed that *V. atypica* improved run time via its metabolic conversion of exercise-induced lactate into propionate, thereby identifying a natural, microbiome-encoded enzymatic process that enhances athletic performance. While other studies reported butyrate as a prominent feature of the athletic microbiota [13, 14, 72, 88, 98, 99], this research implicated propionate as another significant beneficial SCFA. Moreover, these preclinical, proof-of-concept experiments displayed for the first time that beneficial microbes from athletes can be effectively transferred to enhance performance. While performed in animal model, this research is notable as it took an important step from correlative to causal function, implicating athlete microbiomes for broader human health and performance applications.

#### Chronic exercise effects on the human gut microbiota

In a longitudinal research design, Allen et al. [98] reported for the first time that exercise training can modulate the composition and metabolic capacity of the human gut microbiota in previously sedentary individuals. Lean and obese subjects underwent 6 weeks of endurance training with diet controlled, followed by a 6-week washout period [98]. Exercise-induced modulations of the gut microbiota and SCFA were strongly associated with changes in body composition in lean participants and VO_2_max in obese participants, independent of diet. After the washout period, exercise-induced changes in the microbiota were largely reversed once exercise training ceased. This study supports the notion that gut microbiota composition is linked to exercise status and that exercised-induced changes may be temporary and require continual stimulus [100]. Of note, the exercise induced shifts in SCFA-producing taxa (*Faecalibacterium* species [spp.] and *Lachnospira* spp.) and genetic machinery (butyrate-regulating gene, BCoAT) were more substantial in lean versus obese participants. This connection is also supported by the observed shifts in metabolic capacity of the gut microbiota which may be transient and likely dependent on repeated exercise stimuli.

Taniguchi et al. [101] evaluated whether endurance exercise modulates the gut microbiota in elderly subjects, and whether these changes are associated with host cardiometabolic phenotypes. In a randomized crossover trial, 33 elderly Japanese men participated in a 5-week endurance exercise program. 16S rRNA gene-based metagenomic analyses revealed that the effect of endurance exercise on gut microbiota diversity was not greater than interindividual differences, whereas changes in alpha diversity indices during intervention were negatively correlated with changes in systolic and diastolic blood pressure, especially during exercise. Microbial composition analyses showed that the relative abundance of *Clostridioides difficile* decreased, whereas that of *Oscillospira* increased during exercise compared to the control period. The changes in these taxa were correlated with the changes in several cardiometabolic risk factors. These findings indicate that the changes in gut microbiota were associated with cardiometabolic risk factors, such as systolic and diastolic blood pressure. It appears microbial SCFAs influence blood pressure by interacting with host SCFA receptors [102]. In another study with elderly subjects, Morita et al. [103] examined the effect of a 12-week exercise intervention on the composition of intestinal microbiota in healthy women. The relative abundance of intestinal *Bacteroides* increased in subjects that completed aerobic exercise. In addition, the increases in *Bacteroides* following the exercise intervention were positively associated with increases in a 6-min walk test. It is widely accepted that lower levels of *Bacteroides* are associated with the higher prevalence of obesity and metabolic syndrome and that *Bacteroides* species may help in suppressing metabolic dysfunction [79, 104]. However, *Bacteroides* sometimes correlate with higher BMI and a Westernized diet [105]; therefore, the increased relative abundance observed after the 12-week exercise intervention may be due to its ability to shift substrates compared to other taxa that more reproducibly decline with a high-fat diet (i.e., *Bifidobacteria*).

More recently, Liu et al. [106] conducted a well-controlled exercise intervention in 39 prediabetic, medication-naive overweight men with a comprehensive metagenomics and metabolomics analysis. Subjects maintained their normal dietary routine and were randomized into either a control group (no exercise; *n* = 19) or a supervised high-intensity exercise program (*n* = 20) consisting of 70 min combined aerobic and resistance interval training 3 times per week. Despite the overall metabolic benefits of the intervention, ~ 30% of subjects responded poorly to exercise in terms of improvement in glycemic control and insulin sensitivity. Those that did respond showed a remarkable decrease in fasting insulin and Homeostatic Model Assessment of Insulin Resistance index values (− 42.70% and − 49.60%, respectively). Upon investigating the gut microbiota there was a clear segregation in compositional and functional changes between exercise responders and non-responders, accompanied by distinct alterations in microbial metabolites. Specifically. responders displayed greater gene expression of functional pathways generating SCFAs and breaking down BCAAs. These may have been related to the positive findings in glucose metabolism as the rise of BCAA has been associated with insulin resistance [107]. There was also a trend in changes of the metabolites of amino-acid catabolism (BCAAs and aromatic amino acids) and carbohydrate fermentation that was consistent with the altered patterns of genes encoding for associated metabolic enzymes. Conversely the microbial profiles of non-responders after the exercise intervention shared more similarity with those of the sedentary controls, suggesting a maladaptation of gut microbiota in these individuals. Similar to the findings from Barton et al. [13] in professional rugby athletes, pathways involved in DNA replication and amino acid metabolism were preferentially enhanced in responders. Moreover, genes related to glycan biosynthesis and lipid metabolism were elevated in responders. Although there was no obvious difference in baseline microbial structures between responders and non-responders, Liu and colleagues were able to establish a model based on a machine-learning algorithm from baseline microbiome signatures which accurately predicted the exercise outcomes with respect to glycemic control and insulin sensitivity. This raised the possibility of screening for individuals with high likelihood of exercise resistance using gut microbiota, so that personalized adjustments can be implemented in time to maximize the efficacy of exercise intervention.

To examine the potential causal relationship between the differentially shaped microbiotas, Liu et al. [106] then transplanted conventional antibiotic-treated mice with the microbiota from two responders and two non-responders from the above experiment. A substantial improvement in mice gavaged with microbiota from responders was mimicked in glycemic control and insulin sensitivity, in contrast to the lack of change in mice colonized with microbiota from non-responders. Together, the results from both the human and animal studies indicate that exercise may impose a differential impact on the composition and function of the gut microbiota across individuals. While future research is warranted, this study raised the possibility that the makeup of the gut microbiota may be a determiner for the efficacy of exercise (i.e., responders vs non-responders) and that targeting the gut microbiota could maximize the benefit of exercise. It may have been that exercise amplified subtle differences of the gut microbiota at baseline by remodeling the intestinal microenvironment (such as inflammatory and oxidative status and local immunity) critical for microbial growth and interaction, which ultimately lead to a divergent response of glycemic control to exercise intervention. Finally, these findings further reinforce the notion that the functional capacity of gut microbiota, as assessed by metagenomics and metabolomics, can be significantly altered without major shifts in its community structure, and that changes in host phenotype may be more dependent on the metabolic capacity and metabolites of the microbiota, instead of the composition per se.

In the longest exercise intervention to date, Kern et al. [108] investigated the effects of regular aerobic training of different intensities and modalities with similar exercise energy expenditure on gut microbiota over a 6-month period. A total of 88 sedentary overweight/obese subjects were randomized into four arms, including habitual living (control), active commuting by non-motorized bicycle, leisure-time exercise of moderate intensity, or vigorous intensity exercise. Beta diversity changed in all exercise groups compared to control, with participants in the vigorous intensity group showing decreased heterogeneity. Further, the vigorous exercise group experienced a greater increase in alpha diversity at 3 months compared to control. More intense exercise may be needed to induce change in the gut microbiota in sedentary, overweight/obese subjects. In a study of acute exercise, both high intensity interval and moderate continuous training affected the gut microbiota in insulin resistant, sedentary individuals following a 2-week exercise intervention [109]. Specifically, *Bacteroidetes* increased and the *Firmicutes/Bacteroidetes* ratio decreased. This outcome has relevance to athletes as the increase in *Bacteroidetes* plays an essential role in the metabolic conversions of complex sugar polymers and degradation of proteins [110]. There was also a decrease in *Clostridium* and *Blautia* genera. *Clostridium* plays an important role in whole-body immune responses, while *Blautia* purportedly increases the release of proinflammatory cytokines [111]. Interestingly, colonic glucose concentrations associated positively with *Bacteroidetes* and inversely with *Firmicutes* phylum, the *Firmicutes/Bacteroidetes* ratio, and *Blautia* genus. In addition, lower abundance of *Blautia* genus was associated with better whole-body insulin sensitivity. These results highlight the importance of intestinal substrate uptake on the whole-body and changes, especially in glucose uptake, might have a positive effect on the gut microbiota.

Finally, in an observational study, Keohane et al. [99] explored the gut microbiota response of four well-trained ultra-endurance male athletes to prolonged, high intensity trans-oceanic rowing, describing changes in microbial diversity, abundance, and metabolic capacity. Serial stool samples were obtained from the athletes for metagenomic whole-genome shotgun sequencing to record microbial community structure and relevant functional gene profiles pre-race, mid-race, race-finish, and 3 months post-race after a continuous, unsupported 33-day, 5000-km transoceanic rowing event. Alpha diversity increased throughout the ultra-endurance event and was evident as early as day 17 in the race. This increase occurred independent of any change in cardiorespiratory fitness, with VO_2_max similar pre- and post-race. Variations in taxonomic composition included increased abundance of butyrate producing species and species associated with improved metabolic health and improved insulin sensitivity.

The functional potential of bacterial species involved in specific amino and fatty acid biosynthesis also increased. Specifically, the gene expression of functional metabolic pathways involved in L-isoleucine and L-lysine production increased, which play an important role in reducing muscular fatigue and damage during strenuous exercise [112]. Microbial-derived lysine may also contribute to the body protein pool in humans [113]. Changes in essential amino acid availability influence hematopoiesis, which in turn may increase oxygen carrying capacity and cardiorespiratory fitness [112]. Many of the adaptations in microbial community structure and metaproteomics persisted at 3 months follow up.

#### Summary of the effect of exercise on the gut microbiota

Overall, the mechanisms by which physical activity may promote a rich bacterial community and increased functional pathways have not been fully elucidated but likely involve a combination of intrinsic and extrinsic factors. For example, physically active individuals are more likely to be exposed to their environmental biosphere (e.g., time spent outdoors) and follow an overall healthy lifestyle and, consequently, harbor a richer microbiota. Simultaneously, intrinsic adaptations to endurance training, such as decreased blood flow, tissue hypoxia, and increased transit and absorptive capacity can lead to changes in the GI tract [114, 115]. Changes in GI transit time have been reported to affect the pH within the colonic lumen which could lead to alterations in the composition of the gut microbiota. For instance, longer colonic transit time is associated with decreased gut microbiota diversity, which is paralleled by an increase in pH during transit from the proximal to the distal colon [116, 117]. Repeated bouts of aerobic exercise can increase GI transit time in healthy individuals and middle-aged patients with chronic constipation [118–120]. However, at higher intensities (e.g., above 70% VO_2_max), gastric emptying appears to be delayed [121–124]. Aerobic exercise also increases fecal SCFA concentration which can decrease pH in the colonic lumen [125]. Furthermore, metabolites that are a by-product of exercise and circulate throughout the body (e.g., lactate) may filter through the gut and serve as an energy source for certain bacterial taxa (e.g., *Veillonella*). There is expected competition for nutrients and resources in every ecosystem, including the gut microbiota. Therefore, many of these microbial characteristics may be a result of ‘form fits function’, as communities in the gut are shaped by available resources, as determined by the physiology of their host. These and other potential adaptive mechanisms, such as a change in gut pH, may create an environmental setting that allows for richer community diversity and metabolic functions. Anaerobic capacity and resistance exercise training may also influence community composition, though to date, no work has examined these parameters in relation to gut microbiota.

A single acute bout of prolonged excessive exercise can have a deleterious influence on intestinal function. Intense exercise redistributes blood from the splanchnic circulation to actively respiring tissues [126]. Prolonged intestinal hypo-perfusion impairs mucosal homeostasis and causes enterocyte injury. Intestinal ischemia may result, particularly in the setting of dehydration, manifesting as abdominal cramps, diarrhea, or occasionally bloody diarrhea [127]. This adverse effect is particularly the case in endurance sports [128]. As a result, increased intestinal permeability ensues, thought to be driven by the phosphorylation of several tight junction proteins [129]. These events render the gut mucosa susceptible to endotoxin translocation [130]. Moderate endurance exercise in mice has been associated with a lesser degree of intestinal permeability, preservation of mucous thickness, and lower rates of bacterial translocation along with up-regulated anti-microbial protein production and gene expression in small intestinal tissue (α-defensin, β-defensin, Reg IIIb and Reg IIIc) [131]. Together these changes might help mitigate the effects of stress-induced intestinal barrier dysfunction. In humans, physical activity can improve gastrointestinal symptoms in subjects with irritable bowel syndrome [132]. Collectively, these outcomes are evidence of a differential and dose-response effect of exercise on gut health, with the underlying mechanisms yet to be fully explored in healthy humans.

The current body of research supports the role of exercise as an important behavioral factor that can affect qualitative and quantitative changes in the gut microbial composition and function with benefits to the host. Although these changes may not occur in a similar fashion across individuals and may also depend on baseline characteristics of both the microbiota and host. However, based on the current body of research, exercise appears to enrich microbiota diversity, stimulate the proliferation of bacteria which can modulate mucosal immunity, improve barrier functions, and stimulate bacteria and functional pathways capable of producing substances that protect against gastrointestinal disorders and improve performance (i.e., SCFAs). Indeed, exercise may be an important intervention to alter gut microbiota composition and restore gut symbiosis [100]. However, excessive and/or prolonged high-intensity exercise may not impart these effects. Notably, certain taxa may be enriched in athletes such as the lean phenotype-associated *A. muciniphila,* and propionate producing *Veillonella* (via metabolism of lactate). In addition, higher diversity of microbiota composition was associated with lean phenotypes compared to that of obese individuals. It is likely that the diverse, metabolically favorable intestinal microbiota evident in the elite athlete is the cumulative manifestation of many years of high nutrient intake and high degrees of physical activity and training throughout youth, adolescence, and during adult participation in high-level sports [133]. Future areas of gut microbiota research in relation to athletes and exercise is presented in Table 3.

**Table:** 

**Key Points 2 – Exercise and gut microbiota**	
• Higher cardiorespiratory fitness (as measured by VO_2_peak) appears to be correlated positively with increases in microbial diversity and metabolic function, and increases in the SCFA butyrate.	
• Exercise training can modulate the composition and metabolic capacity of the human gut microbiota in sedentary individuals.	
• Changes in host phenotype may be more dependent on the metabolic capacity and metabolites of the microbiota, instead of strictly microbial composition.	
• Changes in the gut microbiota exerted by exercise seem to depend on the physiological state of the individual.	
• Gene content/diversity in the gut may be a better predictor of physiological states compared to species richness.	
• Exercise may promote a rich bacterial community-induced shift in SCFA-producing taxa by providing selective advantage for the colonization of certain microbes, including *A. muciniphila* and *Veillonella*.	
• Exercise induced shifts in metabolic capacity of the gut microbiota may be transient and likely dependent on repeated exercise stimuli.	
• Prolonged excessive exercise has a deleterious influence on intestinal function, including increased intestinal permeability.	
• Nearly all studies included in this review have shown positive correlations between gut taxa and exercise. Overall exercise appears to enrich microbiota diversity, stimulate the proliferation of bacteria which can modulate mucosal immunity, improve barrier functions, and functional pathways capable of producing substances (e.g., butyrate and propionate) that can increase performance and health.

**Table 3:** Future areas of gut microbiota research in relation to athletes and exercise

Current gaps in knowledge:^**a**^	Recommendation(s) for future studies to address:
1. Athletic-focused studies have assessed the taxonomic composition of the gut microbiota, while placing less emphasis on functional capacity.	• The functional capacity of gut microbiota, as assessed by metagenomics and other omic-driven techniques (e.g., metabolomics), can be significantly altered without major shifts in community structure. Effects on the host may be more dependent on the metabolic capacity and metabolites of the microbiota, instead of the composition per se. Therefore, investigators should incorporate functional assessment of the gut microbiota.
2. At present it is unclear how exercise affects the gut microbiota over long durations (i.e., > 6 months).	• Assess the effect of exercise on the gut microbiota, as well as the gut microbiota in athletes over longer periods of time.
3. Little research is available directly comparing the potential differences in the gut microbiota between athletic disciplines, exercise routines, training loads, and physical fitness status.	• Examine the potential differences in the gut microbiota between these factors in both cross-sectional and longitudinal investigations.
4. There is limited research on how environmental factors affect the gut microbiota in athletes.	• Explore the effect of temperature, humidity, pollen/allergens, and other relevant environmental factors in relation to the gut microbiota in athletes.
5. There is limited research available on how intrinsic adaptations to exercise impact the gut microbiota.	• Investigate how such factors as decreased blood flow, tissue hypoxia, and increased/decreased transit and absorptive capacity in the GI tract can lead to changes in the gut microbiota.
6. Some research is available showing prolonged excessive exercise has a detrimental influence on intestinal function.	• Investigate how prolonged, excessive exercise impacts intestinal function (i.e., increased intestinal permeability) and the gut microbiota.
7. Currently the gut-brain axis remains generally unexplored in athletes.	• Explore the gut-brain axis in athletes, as well as the impact of exercise in sedentary individuals.
8. Currently the role of *Akkermansia muciniphilia* has not been fully elucidated in humans, although appears to be more present in athletes compared to non-athletes.	• Continue to investigate the role *Akkermansia muciniphilia* plays in the gut microbiota and its functional impact on metabolism.
9. In relation to obesity, some athletes who may be defined as physically active using common criterion may not necessarily be healthier based on BMI. There is limited research comparing these athletes to sedentary individuals matched for BMI.	• Investigate this comparison at the obese classification. Findings from this research could provide important data in connection with the pathogenesis of obesity and the gut microbiota.
10. The influence of energy stores (obese or lean state) and energy intake (positive or negative energy balance) on ability to alter the gut microbiota remain unclear.	• Gut microbiota research in athletes with high, as well as low energy consumption requires further investigation. • Research in those implementing caloric reduction with exercise for weight loss is also needed.
11. The functional capacity of the athletic gut microbiota is not fully understood, particularly the relationship to the significant energy demands and tissue adaptation that occurs during intense exercise and elite sport.	• Intervention-based studies should be conducted to further delineate this relationship. • Results from such research will be important and may provide further insights into optimal therapies to influence the gut microbiota, and its relationship with health and disease as well as athletic performance.
12. Few studies have focused on the impact that voluntary exercise has on gut microbiota.	• More animal research should be conducted looking at the difference between forced vs. voluntary exercise in relation to exercise volume.
13. Some animal research reported exercise induces more effective changes in the microbiota in juvenile rats compared to adult rats.	• Research in animal models, as well as humans, should examine the effect of exercise on the gut microbiota comparing different age classifications.
14. Currently a few preclinical experiments have shown that beneficial microbes from athletes can be effectively transferred to enhance performance.	• Research in animal models should continue to test the functions of these microbes expressed in athletes (e.g, *Veillonella*) and explore their implications for broader human health and performance applications.
15. Limited research has shown that exercise-induced gut microbiota changes may be temporary and require continual stimulus.	• More research is needed investigating the temporal effect of exercise stimuli on the gut microbiota and its functional capacity, both at the acute and chronic level.
16. Limited research has reported a segregation in compositional and functional changes between exercise responders and non-responders, accompanied by distinct alterations in microbial metabolites.	• Replication of these findings and further investigation of the possibility that the makeup of the gut microbiota may be a determiner for the efficacy of exercise (i.e., exercise responders vs non-responders) • Explore the possibility of targeting the gut microbiota of ‘non-responders’ to increase the benefit of exercise.
17. To date, no study in athletes has addressed RED-S^b^ syndrome in relation to the gut microbiota. Moreover, little is known on the effects of energy reduced diets in athletes looking to healthfully reduce bodyweight and/or improve body composition.	• Dissect the impact of restricted energy consumption and/or increased energy expenditure on the gut microbiota in athletes.
18. The effects of high-protein (without concurrent high-fat) consumption on gut bacteria are not well studied. This also includes the effects of high-protein and high-fiber intake.	• Investigate the effect of higher protein consumption on the gut microbiota, particularly in the context of lower fat and higher fiber intakes. • The types and amounts of fats consumed in conjunction with protein should be investigated in the overall effect on the gut microbiota. • Examination of the effect of prebiotics and probiotics in conjunction with increased protein intake.
19. Protein intake appears to be a strong modulator of gut microbiota diversity, however there is little research examining the effect of protein supplementation.	• More research needs to be conducted investigating the effect of protein supplementation, such as whey and vegetable-based proteins, on the gut microbiota.
20. Early research has reported changes in the gut virome from protein supplementation, suggesting virus particles from whey protein transmit to the gut from consumption.	• The effect of the gut virome on the gut microbiota and host requires further investigation, particularly in relation to food and supplement consumption.
21. The genus *Prevotella* has been reported to be positively associated with both health and disease states.	• More studies in humans are needed to better understand *Prevotella*’s role in athletes, as well as its role in disease. For this, more in-depth metagenomic studies will be required to reveal the health- or disease-modulating properties of *Prevotella*, particularly at species and functional level.
22. Little is known about exercise-nutrient interactions that underpin adaptation and performance.	• Further research is needed to determine the synthesis kinetics and clinical consequence of microbial by-products during increased nutritional status and metabolic demands during exercise. • Determine if specific nutrient recommendations aimed at improving performance can be made by enhancing certain metabolites during exercise and recovery. This includes limiting those that produce toxic metabolites that may worsen the consequences of exercise stress.

^a^Gaps observed in studies from the current review

^b^Relative Energy Deficiency in Sports

### The effect of athletic diet on the gut microbiota

In researching the human gut microbiota, it is difficult to examine exercise and diet separately. This relationship is compounded by changes in dietary intakes often associated with physical activity (e.g., increased protein intake in resistance trained athletes or carbohydrate intake in endurance athletes and increased total energy and nutrient intake in general). Athletes often consume a diet that differs from the general population with implications on the composition of the gut microbiome.

Diet is an established modulator of gut microbiota composition, with significant alterations reported within 24 h of a dietary change [134]. This ability to rapidly change has implications in research design for the timing of measurements in exercise studies, as does dietary composition. Indeed, various food components, dietary patterns, and nutrients all have the potential to substantially alter the growth of different gut microbial populations. Medication and diet are principal environmental factors that influence gut microbiota composition according to large-cohort studies [135, 136]. The gut microbiota is an important factor that shapes both energy harvest and storage through metabolism of proteins and production of several metabolites including SCFAs, ammonia, sulfur-containing metabolites such as hydrogen sulfide and methanethiol, and neuroactive compounds such as tryptamine, serotonin, phenethylamine, tryptophan, and histamine [137, 138]. Moreover, the gut microbiota can also synthesize de novo amino acids and is involved in the utilization and catabolism of several amino acids originating from both alimentary and endogenous proteins. These amino acids can serve as precursors for the synthesis of other metabolites produced by the microbiota including SCFAs [139]. Animal studies have revealed communication between the gut microbiota and muscle, in which gut microbiota can affect muscle energy homeostasis by interfering with fat deposition, and lipid and glucose metabolism through various metabolites including SCFAs and secondary bile salts [17]. Broadly, athletes consume higher energy diets compared to sedentary individuals and are often encouraged to consume a diet high in carbohydrate and protein and lower in fat [140]. During training and competition, fiber intake may be reduced to avoid potential GI issues including gas and distension [141]. Importantly athletes’ dietary plans often account for macro- and micronutrient needs, hydration, the timing of nutrients, and dietary supplements, but rarely is the health of the gut microbiota considered [140]. Here we describe the influence of total energy intake and the principal macronutrient classes (protein, carbohydrate, and fat) on the gut microbiota.

#### Energy intake

The GI tract represents the interface between ingested nutrients and the host where energy is effectively extracted. In healthy adults, ~ 85% of carbohydrates, 65–95% of proteins, and nearly all fats are absorbed before entering the large intestine [142]. Consequently, indigestible carbohydrates and proteins that enter the colon represent between 10 and 30% of total ingested energy [143, 144]. If not for the colonic microbiota, these nutrients would generally be eliminated via the stool without further absorption due to the limited digestive capability of the human large intestine [142]. Therefore, the gut microbiota plays an important role in energy extraction and, in turn, can be influenced by the composition of the diet and the amount of energy entering this environment [145]. In relation, the gut microbiota produces and releases an enormous array of compounds which may act upon host tissues modulating appetite, gut motility, energy uptake and storage, and energy expenditure [146, 147]. Riedl et al. [148] estimated that for an average 90 kg male, the biomass of bacteria in the gut could be expected to contribute anywhere from 7 to 22% of daily adult human caloric turnover (based on 2000 kcal [kcal] per day). Clearly, the gut microbiota-host interaction can affect energy balance which has implications for weight gain or loss and body composition [149, 150].

Strong evidence exists to support the role for the gut microbiota in energy balance by contributing to host digestive efficiency [151]. Studies of lean and obese mice indicate that the gut microbiota affects energy balance by influencing the efficiency of calorie harvest from the diet and how this harvested energy is used and stored. For example, studies of germ-free mice (so called ‘gnotobiotic mice’) have provided important insights into the role the gut microbiota plays in energy homeostasis. Gnotobiotic mice are inefficient at processing food, yet when colonized with conventional mouse gut biota they gain weight by increasing their energy stores [152]. This weight gain occurs even when decreasing energy intake by 30% and increasing energy expenditure by 30%, compared to mice who remained germ-free [153]. These results implicate the gut microbiota as an energy harvester, significantly affecting nutrient absorption by extracting energy from dietary substances.

To examine the impact of energy consumption on the gut microbiota, rats fed a high-energy dense diet rapidly altered their gut microbiota with increases in *Firmicutes/Bacteroidetes* ratio and in pro-inflammatory *Proteobacteria* proliferation compared to those consuming a low-energy diet [154]. Moreover, the high-energy diet increased circulating pro-inflammatory LPS. However, the impact of energy consumption on, and the ultimate extraction by, the gut microbiota is deeply intertwined with composition of the ingested diet. For example, obese mice fed a low saturated fat, high fruit and vegetable diet can take on microbiota characteristics of lean mice [63]. Moreover, mice consuming this diet regardless of lean or obese state gained less fat mass compared to lean and obese mice fed a high-saturated fat, low fruit and vegetable diet, typical of a Westernized diet.

In terms of human research there are few studies that have examined the effect of energy intake and energy expenditure on the gut microbiota. The majority of this research has been conducted in relation to the study of obesity, weight loss, and malnourishment in children. Generally, when comparing obese and lean individuals, both the diversity of the gut microbiota and the ratio of *Bacteroidetes* to *Firmicutes* is decreased in obese individuals [145]. Similar findings have been reported in relation to gene richness and altered metabolic pathways [155]. However, the composition of the gut microbiota does appear to be sensitive to caloric balance as noted in subjects studied before, during, and after weight loss [38]. Furthermore, improved gene richness has been reported during weight-loss and weight-stabilization interventions in obese and overweight individuals [156]. What remains unclear is the influence of energy stores (obese or lean state) versus the impact of energy intake (positive or negative energy balance) on ability to alter the gut microbiota. In a carefully monitored inpatient crossover feeding trial, Jumpetz and colleagues [157] examined how gut bacterial community structure is affected by two distinct caloric loads (2400 vs 3400 kcal/day) with a similar nutrient profile (24% protein, 16% fat, and 60% carbohydrates) and dietary energy harvest in 12 lean and 9 obese individuals. The higher caloric load was positively correlated with the relative abundance of *Firmicutes* species and negatively correlated with the relative abundance of *Bacteroidetes* species in both lean and obese humans. In lean individuals, these changes were associated with an increased energy harvest of approximately 150 kcal. This finding suggests that the microbiota is responsive to energy balance (degree of overfeeding) as well as actual adiposity. It may be that the gut “senses” alterations in nutrient availability and subsequently modulated the nutrient absorption. Regardless, these results show that the nutrient load is a key variable that can influence the gut community structure. In rugby athletes with high energy consumption (median intake of 4449 kcals per day), gut microbial diversity was significantly greater compared to age and BMI matched sedentary controls (median intake of 2801 kcal per day) [19]. Moreover, in cyclists consuming high-energy, high-carbohydrate diets, abundances of health associated bacteria were high (including *Prevotella* and *Akkermansia*) and less characteristic of Western-associated microbiota [18]. However, it is difficult to remove physical activity influence from this, and gut microbiota research in athletes with high energy consumption requires further investigation.

In contrast to high-energy intake and obesity, even less is known about the gut microbiota in undernutrition [142]. Athletes can have a tremendous energy expenditure often requiring a corresponding increase in dietary intake to maintain energy balance. However, Relative Energy Deficiency in Sports (RED-S) syndrome is present in many athletic disciplines as a result of insufficient energy availability due to insufficient caloric intake and/or excessive energy expenditure [158]. Occurring in both males and females, RED-S possesses a significant health risk. To date, no study in athletes has addressed RED-S in relation to the gut microbiota. Moreover, little is known on the effects of energy reduced diets in athletes looking to healthfully reduce bodyweight and/or improve body composition. Calorie restriction, primarily in animals, can improve the composition and associated metabolism of the gut microbiota, including increasing the relative abundances of probiotic and butyrate-producing microbes [159] and increasing SCFA biosynthesis [160].

In humans, severe calorie restriction as a result of bariatric surgery offers an interesting research model to explore the effect on the gut microbiota [161]. Changes such as reduced abundance of *Firmicutes* post-surgery have been reported [162]. Although it is unclear if these modifications were caused by dietary change or weight loss. Furet and colleagues [163] reported that the *Bacteroides*/*Prevotella* ratio increased within 3 months after surgery and remained stable thereafter. While this ratio was negatively correlated with body weight, BMI, and body fat mass, the correlation was highly dependent on total calorie intake. Other alterations, such as the reduction of lactic acid forming bacteria, indicate a complex effect of severe calorie restriction.

Undernourished children have been observed to exhibit impaired gut microbiota development, with reduced relative abundance of several *Bifidobacterium* and *Lactobacillus* spp. as well as obligate anaerobic SCFA-producing taxa [164]. For instance, a sample of children living in an urban slum in Bangladesh with either moderate acute malnutrition or severe acute malnutrition had a gut microbiota that was ‘immature’; meaning discriminatory taxa in their gut communities were more similar to younger rather than age-matched healthy individuals from the same location [165]. This ‘immaturity’ was greater in those more severely malnourished with probable physiologic, metabolic, and immunologic consequences [165]. This has led to the proposal that disrupted microbiota development impairs healthy bone and muscle growth during infancy [166]. To explore the association between nutrition and the gut microbiota during infancy, Charbonneau et al. [166] colonized young germ-free mice with the fecal microbiota of a growth-stunted Malawian infant. These animals were fed a representative Malawian diet with or without a bioactive substance in breast milk (purified sialylated bovine milk oligosaccharides). Treatment with the milk oligosaccharides produced microbiota-dependent growth promotion, including lean body mass gain, changed bone morphology, and altered liver, muscle and brain metabolism. These effects were also documented in gnotobiotic piglets using a similar design showing a greater ability to utilize nutrients from the diet [166]. These preclinical models indicate a causal, microbiota-dependent relationship between nutrition and growth promotion which may have implications for younger athletes.

Various studies have explored the gut microbiota of anorexia nervosa patients with the majority of them being characterized by heterogeneity in the methodology and small sample sizes (for review see: [167]). Several studies of anorexia nervosa patients have reported decreased abundances of the butyrate producing *Roseburia* in combination with reduced butyrate levels and lower microbial diversity and taxa abundance compared to healthy controls [168–170]. During an in-patient, medically supervised weight gain study in anorexic individuals, microbial richness increased, however perturbations in intestinal microbiota and SCFA profiles, in addition to several gastrointestinal symptoms, did not recover during the subject’s in-patient stay [169]. Future studies will be needed to dissect the impact of restricted energy consumption and/or increased energy expenditure on the gut microbiota in athletes.

Overall, energy balance is an overlooked factor in relation to the athletic gut microbiota. Not only is this relevant to improving performance, but also addressing the health status of those affected by RED-S. Different dietary patterns affecting macronutrient consumption can alter the composition of what enters the large intestine where there is the greatest density of gut microbes. This has a tremendous impact on the human body’s ability to extract and utilize energy from the diet. Moreover, it is difficult (if not impossible) to solely investigate the impact of total energy consumption on the composition of the gut microbiota without considering dietary variability such as the major dietary macronutrient classes.

#### Protein

Despite the difficulties of studying macronutrient effects in isolation, there is evidence to support the assertion that dietary protein (and fat) consumption elicit both compositional and functional changes to the gut microbiota [171]. David et al. [134] showed a rapid shift in gut microbial community composition and increased populations of *Alistipes*, *Bilophila*, and *Bacteroides* after consuming a high-fat/protein diet for 5 days and these changes were thought to be a result of increased bile secretion. Changes to the gut microbiota have also been documented when dietary protein is increased: *Bacteroides* spp. are highly associated with animal proteins, whereas *Prevotella* spp. are highly associated with increased intakes of plant proteins [172]. Intervention studies have demonstrated that high-protein diets (animal protein) reduced fecal butyrate concentrations and butyrate-producing bacteria such as *Bifidobacteria* spp., *Roseburia* spp., and *E. rectale* [173–175]. Fecal concentrations of potentially damaging N-nitroso compounds increase markedly in volunteers who consumed a high-protein, low-carbohydrate diet [175]. Furthermore, a study of five male volunteers consuming high intakes of animal protein showed that fecal sulfide production is related to meat intake [176]; notably, hydrogen sulfide is a compound associated with ulcerative colitis [177]. Ma et al. [178] suggested that excessive protein intake or an unsuitable ratio of protein to protein-fermenting bacteria, could potentially produce adverse effects on health.

The partitioning of individuals into so-called ‘enterotypes’ (stratifying global microbiome variation into a few categories, reviewed in [179]) has been suggested to be driven by whether their primary dietary patterns include high complex carbohydrate (*Prevotella*) or high-fat/protein (*Bacteroides*) consumption [172]. This categorization has been criticized as an oversimplification, obscuring potentially important microbial variation, and may not be appropriate for the athletic population [180, 181]. For example, the *Bacteroides* enterotype has been suggested to most strongly be correlated with frequent consumption of animal protein and saturated fat. However, the effects of high-protein consumption (without concurrent high-fat) on gut bacteria are not well studied but of increasing importance given the current popularity of high-protein diets, especially in athletes. In professional rugby players, distinct compositional and functional microbial characteristics, including increased alpha diversity, enhanced microbial production of SCFAs, and greater metabolic capacity are evident in the gut [13, 19]. These microbial features not only positively correlate with the athletes’ levels of physical activity, but also the quantity of dietary protein consumed. In many athletic disciplines, as well as recreational exercise, protein supplementation (e.g., whey protein) provides a sizeable proportion of athletes’ daily protein intake [19]. Clarke et al. [19] reported microbiota diversity indices correlated positively with protein intake and serum creatine kinase indicating that diet and exercise are both drivers of biodiversity in the gut. The protein and microbiota diversity relationship is further supported by a positive correlation between blood urea levels (a by-product of diets rich in protein) and microbiota diversity [19]. In contrast, Jang et al. [11] reported that daily protein intake was negatively correlated with alpha diversity in distance runners. The inconsistency of these results compared to Clarke et al. [19] may relate to the nutritional status of the athletes. In addition, the study by Clarke and colleagues [19] met all of the recommended dietary intake requirements, while the athletes in the investigation by Jang et al. [11] had insufficient carbohydrate and dietary fiber intake (compared to standard macronutrient distribution ranges). It seems that high-protein diets may have a negative impact on gut microbiota diversity for athletes in endurance sports who consume lower amounts of energy, carbohydrates, and dietary fiber, while athletes in resistance sports that follow a high-protein, low-carbohydrate, and high-fat diet demonstrate a decrease in SCFA-producing commensal bacteria. Long-term diets have been linked to certain compositional clusters in the gut microbiota: protein and animal fat are associated with *Bacteroides* enrichment and simple carbohydrates with *Prevotella* enrichment [172]. Excessive fermentation of dietary protein in the GI tract is generally considered detrimental given the production of toxic by-products such as amines, phenols, indoles, thiols, and ammonia [182, 183]. In contrast, feeding of whey protein to mice attenuated some negative effects on the composition of the gut microbiota composition including increasing *Lactobacillaceae/Lactobacillus* and decreasing *Clostridiaceae/Clostridium* [184, 185]. Further, whey protein has been associated with reductions in body weight and increased insulin sensitivity in the past, and is frequently a major component of the athlete diet, particularly in strength and power sports [186, 187].

Estaki et al. [14], reported total protein intake was a major contributor to increased beta diversity (the ratio between regional and local species diversity) at each taxonomic rank tested in 39 healthy adults with varying cardiorespiratory fitness levels. A strong association was evident between protein intake and *Bacteroides* and, in particular, *Ruminococcaceae* and *Lachnospiraceae*, two of the most abundant families in human gut environments [188], in explaining community diversity. These saccharolytic organisms persist in fibrolytic gut communities and are considered an important component of a healthy gut microbiota, while their depletion has been observed in inflammatory bowel disease patients [189, 190].

In comparing athletes to both high and low BMI non-athlete controls, Barton et al. [13] reported a greater number of pathways correlating to specific macronutrients within the control participants suggesting a shift in the dynamics of varied metabolic functions. The impact of the athletes’ increased protein intake compared to both control groups was evident in the metabolomic phenotyping results. Metabolites derived from dietary protein (trimethylamine N-oxide, carnitines, trimethylamine, 3-Carboxy-4-methyl-5-propyl-2-furanpropionic acid, and 3-hydroxy-isovaleric acid), muscle turnover (creatine, 3-methylhistidine, and L-valine), vitamins and recovery supplements (glutamine, lysine, 4-pyridoxic acid, and nicotinamide), as well as phenylacetylglutamine (a microbial conversion product of phenylalanine) were increased in athletes [191].

Investigating the gut microbiota of cyclists, Petersen et al. [18] reported an increased abundance of *Prevotella* which positively correlated with a number of amino acid and carbohydrate metabolism pathways, including BCAA metabolism. High levels of BCAAs (leucine, isoleucine, and valine) can attenuate exercise-induced muscle fatigue and promote muscle-protein synthesis [192]. While there is strong evidence showing that BCAAs do not enhance exercise performance [192, 193], they may reduce central fatigue [194] and attenuate muscle damage during prolonged exercise [195]. Since BCAAs are not produced by the human body and need to come from the diet, having a gut microbial community that contains *Prevotella spp*. to either synthesize BCAAs or alternatively influence other microbes to produce these amino acids would be highly beneficial to athletes who require a rapid recovery from intense exercise.

In cross-country runners, the effect of 10 weeks of protein supplementation (10 g whey isolate and 10 g beef hydrolysate per day) consumed daily decreased SCFA-producing bacteria while increasing bacteria with proteolytic activity in the microbiota without affecting SCFAs, ammonia, or fecal pH of endurance athletes [196]. The amount of additional dietary protein was small but yielded a significant 17% increase in dietary protein for these athletes. Specifically, protein supplementation increased the abundance of the *Bacteroidetes* phylum and decreased the presence of health-related taxa including *Roseburia, Blautia*, and *B. longum*. In contrast to Clarke et al. [19], no changes in compositional microbiota diversity were detected after the ten-week intervention, which may relate to the low percentage of protein intake. Increases in dietary protein can increase the amounts reaching the colon, where they are metabolized by colonic microbiota, leading to changes in microbiota populations and in microbial metabolites [178]. The difference between these two studies may also be due to differences in analyses, as Clarke et al. [19] only assessed the V4 region of the gene, while the present study used the V3 and V4 region (see Table 4). Balancing the protein/carbohydrate ratio with prebiotics when protein intake is elevated [197], or accompanying the intake of protein supplements with probiotics, could be future strategies to mitigate the observed or anticipated gut microbiota shift [198].

**Table 4:** Recommendations for future gut microbiota research in athletes and exercise interventions

Observed weaknesses:	Recommendation(s):
1. Current microbiota sequencing methods rely heavily on 16S rRNA amplicon-based methodology which detects up to genus level with minimal capability for species-level detection.	• Investigators are encouraged to utilize other methodology, such as shotgun metagenomics for its expanded taxonomic range, strain-level resolution, and identification of other microorganisms (i.e., archaea, viruses, and fungi, etc.). • Omic techniques, such as metatranscriptomics, metaproteomics, and metabolomics, should be integrated with compositional data to provide a ‘functional’ readout of the microbiome providing data on the metabolic interplay between the host, diet, exercise, and the gut microbiota. • For targeted genomic approaches specific primers for quantitative PCR can be effectively used.
2. Primer selection and hypervariable regions of 16S rRNA (i.e., V1-V9) influence the observed microbial community.	• When using 16S rRNA amplicon-based sequencing it may be better to sequence a single variable region (V4 is mostly used in human gut microbiome studies) with reads that (almost) entirely overlap to reduce errors [235].
3. Several studies report less stringent inclusion/exclusion criteria and did not fully account/control for confounding variables.	• When outlining participant criteria, investigators should pay particular attention to important factors that may confound results such as medication use (e.g., antibiotics, bile acid sequestrants, metformin, etc.), dietary supplement use (e.g., prebiotics and probiotics), age, sex, ethnicity, geographical location, etc. • To control for confounding effects investigators should collect more detailed data on such factors as dietary intake, training history, level of physical fitness, gastrointestinal function (e.g., gastrointestinal symptoms rating scale [236]), fecal pH, fecal form (i.e., Bristol stool scale, [237]), etc.
4. The majority of studies in the present review did not report power and sample size calculations.	• Many studies in this field are likely underpowered. Therefore, investigators are encouraged calculate and report sample size estimations when appropriate.
5. Several studies in the present review did not provide adequate detail on sample collection and processing. Both of these factors can significantly affect the analysis and ultimately the results of the study.	• While there is no one method, investigators should provide detail on these procedures and ensure these methods are the same across samples. • Time that samples are held at room temperature should be greatly minimized (i.e., < 24 h without preservative) and kept consistent across samples [238, 239]. • Homogenization of the whole stool sample may provide a more uniform sample. This method has been shown to reduce the variation in both the amount of DNA extracted and the relative abundance of bacterial taxa [240].
6. Similar to issue #5, several reports did not provide adequate detail in regard to sample transportation and storage.	• Investigators should provide detail on these procedures and ensure these methods are the same across samples. • The gold standard for microbial storage is freezing samples at − 80 °C [241]. • Investigators are encouraged to keep freeze-thaw cycles minimal as they affect reproducibility [242].
7. While not a weakness per se, feces is the only sample material used in the analyses of the gut microbiota from the studies included in this review.	• Eventually, more in-depth mapping of microbial community structure and function along the length of the GI tract and across gradients (i.e., from lumen to mucosa) should be considered [34].
8. The variation in microbial analysis across different studies can make comparing/contrasting study findings difficult.	• Variation in profiling techniques (e.g., sequencing strategies, platforms, variable regions, sequencing depth, etc.) may act as a confounding variable that can lead to significant differences due entirely to laboratory techniques rather than treatment. While a limitation of the current review, future, more specific reviews should provide deeper discussions on results in the frame of their methodological approaches.

^a^Quality weaknesses observed in studies from the current review

Cronin et al. [199] noted participants consuming whey protein daily experienced a marked alteration in the diversity of their gut virome (a collection of viruses that inhabits the gut environment and affect host cells as well as other commensal organisms [200]) following 8 weeks of oral supplementation. Sedentary subjects (predominantly overweight > 30% body fat) were divided into three groups of 30 subjects: an exercise-only group, a daily whey protein supplementation (30 g per day) group, and an exercise plus daily whey protein supplementation (30 g per day) group. Individuals in the whey protein supplementation-only group experienced a significant increase in the beta diversity of the gut virome. Furthermore, this change was mirrored in the combined exercise and protein supplementation group, suggesting a robust positive effect of whey protein on the taxonomic richness of the gut virome. Specifically, all bacteriophages (bacteria-targeting viruses) increased in the groups receiving whey protein were present in high relative abundance within the whey protein supplement. Therefore, it may be virus particles from whey protein transmit to the gut from consumption. The effect of the gut virome on the gut microbiota and host requires further investigation, particularly in relation to food and supplement consumption.

The source of protein, including its quality and digestibility, may influence the site of fermentation within the gut. Highly digestible proteins, such as whey, can be digested by host enzymes in the proximal intestine, reducing microbial fermentation. Similarly, plant-originated proteins are available for microbial fermentation in a more distal site given incomplete digestion by host enzymes, particularly at a higher protein level. Evidence indicates proteins from vegetable origin have a more marked effect on microbial diversity than animal proteins [201], however investigation in athletes is needed. By selecting dietary ingredients containing protein of rather high digestibility and quality, the amount of dietary protein reaching the large intestine may be diminished, thus limiting the quantity of residual protein available for protein fermenting bacteria. As a consequence, the growth and activity of potential pathogens could be suppressed. These seemingly opposing effects of high-protein diets imply that protein-diet interactions are modulated by factors such as host body composition and exercise intensity. The types and amounts of fats consumed in each of these studies are also likely important for the overall effects on the gut microbiota. For example, a ketogenic diet alters gut microbiota composition leading to an increase in *Akkermansia* abundance. Moreover, *Akkermansia* fed to mice has a positive impact on reducing seizures, providing a potential mechanism for the observed neuroprotective effects of a ketogenic diet [66, 202].

#### Carbohydrates

As a macronutrient class, carbohydrates (including dietary fiber) have a profound effect on the gut microbiota. In comparison to bacteria, humans have much fewer enzymes to break down carbohydrates [203] and what can be digested by these enzymes is absorbed in the small intestine. Dietary fiber passes undigested from the small intestine into the colonic environment [171] and the gut microbiota relies on these fibers for energy, which they target for disassembly with a combined ‘toolkit’ of thousands of enzymes [204]. Therefore, carbohydrates in the form of dietary fiber represent enormous potential for modulation of gut microbiota based upon the chemistry and accessibility of specific dietary fibers to microbial groups.

Increased intake of dietary fiber does not have an overall *Bifidobacterium* increasing effect, however, specific dietary fibers have been shown to selectively increase *Bifidobacteria* abundance [205]. Furthermore, increased intake of dietary fiber has been associated with an increase in gut microbial richness and/or diversity, especially in individuals with reduced diversity [206]. Long-term patterns of dietary fiber consumption can also shape the overall bacterial community type. As previously discussed, enterotype assignment to the *Prevotella* group has been suggested to be associated with high-fiber diets. O’Keefe et al. [207] studied the effects on gut microbiota when fat and fiber content of native African and African American diets were swapped for 2 weeks, such that African Americans consumed high-fiber, low-fat rural African diets and vice versa. While changes in abundances of *Bacteroides* or *Prevotella* genera were not observed in either group, the high-fiber, low-fat diets enriched bacterial genes for butyrate production and decreased genes for secondary bile acid synthesis, emphasizing the importance of identifying functional rather than compositional shifts.

Endurance athletes are well known to follow diets that result in the consumption of high amounts of both simple and complex carbohydrates [208, 209]. This dietary pattern, in combination with the substantial number of hours spent exercising on a weekly basis, led to the hypothesis that endurance athletes are likely to have increased abundance of the bacterial genus *Prevotella* [208]. *Prevotella* is normally found in only a small percentage of healthy individuals in European and American cohorts [1, 2, 20, 210]. Previous microbiome studies have repeatedly identified significant correlations of both diet and geographic location to abundances of *Prevotella* or *Bacteroides*. *Prevotella* is more often found in individuals from certain areas of Asia [211, 212] and rural Africa [213], and this enrichment for *Prevotella* is often reflective of diets high in complex carbohydrates (including high dietary fiber from various sources including fruits and vegetables), egg food items, and high levels of vitamins and minerals [172, 211]. However, *Prevotella* has been noted to be associated with several disease states. For instance, *Prevotella* has been shown to be higher in patients with depression [214], insulin resistance [215], non-alcoholic fatty liver disease [216, 217], hypertension [217], and colon cancer [218]. One potential explanation for this phenomenon is that there are several strains within the *Prevotella* genus that exert pathogenic actions, which could help explain the bi-directional and almost opposing effects that *Prevotella* has shown to have in human health [219]. More studies in humans are needed to better understand *Prevotella*’s role in athletes, as well as its role in disease. For this, more in-depth metagenomic studies will be required to reveal the health- or disease-modulating properties of *Prevotella*, particularly at species and functional level [219].

Nutritional strategies (i.e., avoiding fat and fiber) have been recommended to reduce the risk of GI distress before and during training and competition [220]. These recommendations aim to support rapid gastric emptying, water and nutrient absorption and adequate perfusion of the splanchnic vasculature [221]. However, the lack of complex carbohydrates in elite athletes’ diets may negatively affect the gut microbiota composition and function over time. Many athletes may not be consuming enough fiber that feed commensal bacteria that produce beneficial byproducts for host metabolism and homeostasis [15]. Furthermore, adding fiber, including resistant starch, to high protein diets may help reduce potential negative effects of high protein consumption [222] and may increase fat oxidation [202], further illustrating the importance of consuming adequate dietary fiber for gut and overall health.

Petersen et al. [18] reported increased abundance of *M. smithii* transcripts in professional cyclists using RNA sequencing. *M. smithii* increases the fermentation efficiency of many bacterial taxa in the gut, including those that ferment complex polysaccharides [223]. This effect could benefit athletes because an increase in bacterial fermentation products (such as SCFAs) could be absorbed and utilized by the host. Theoretically, this effect could enhance recovery from intense exercise and possibly race performance. SCFAs can improve skeletal muscle insulin sensitivity [224], reduce inflammation [225], and regulate satiety [226], all of which may contribute to the improvements in body composition observed in this study. Additionally, SCFAs are also energy substrates for numerous tissue types, including the colon [227], adipose [228] and muscle tissues [224], indicating that SCFAs can contribute to enhanced energy harvest from the diet, ultimately providing support to healthy tissue growth and turnover.

Within the SCFAs, distinct clusters (acetic acid, propionic acid, and butyric acid) were observed by Barton et al. [13] to positively correlate with dietary contributors (fiber and protein), while isobutyric acid, isovaleric acid, and valeric acid positively correlated with microbial diversity. The same clusters were observed when positively correlating with individual taxa, in support of purported links between SCFAs and numerous metabolic benefits and a lean phenotype [61–63].

#### Fat

Like protein and carbohydrate, the specific effects of fat on the gut microbiota are difficult to isolate; however, the types of fats consumed appear to be important. In a rodent study, animals fed lard showed increases in *Bacteroides* and displayed signs of metabolic dysfunction. In contrast, animals fed fish oil showed increased levels of lactic acid bacteria and were protected from metabolic dysfunction [229]. In humans, ingestion of an animal-based diet for 5 days, high in fat (69% of total energy) and protein (30% of total energy) and almost entirely void of carbohydrates (including dietary fiber), induced rapid and significant changes in microbial community structure and overwhelmed inter-individual differences in microbial gene expression. Specific alterations included gut bacterial taxonomic shifts and transcriptional responses characteristic of carnivorous mammals, with higher concentrations of bile-tolerant bacteria (presumably due to the extremely high-fat intake known to increase bile acid secretion) [134]. Diets high in fat could interact in various ways with the gut microbiota to facilitate the translocation of bacterial LPS generating chronic inflammation [171]. LPS can be incorporated into lipid micelles formed during fat digestion, and certain gut microbes may be important in regulating this process.

In a short-term feeding study Wu et al. [172] randomized ten subjects into a 10-day high-fat/low-fiber diet (38% fat, 35% carbohydrate, 27% protein) while others were given a high-fiber/low-fat diet (13% fat, 69% carbohydrate, 18% protein). Although specific taxa changes varied between individuals, the high-fat diet slowed intestinal transit time by as much as 3 days. Metagenomic analysis indicated that functional shifts, including greater protein export and lipoic acid metabolism, were also associated with the high-fat diet. Finally, the *Bacteroides* enterotype was most strongly correlated with reports of frequent consumption of animal protein and saturated fat. Similarly, Murtaza and colleagues [230] completed a three-week diet intervention in elite race walkers undertaking intensified training combined with a ketogenic, low-carbohydrate, high-fat diet (LCHF; < 50 g day carbohydrate; 78% energy as fat; 2.1 g/kg/day protein) and reported increased relative abundance of *Bacteroides* and *Dorea* and reduced *Faecalibacterium*. In comparison to high or periodized carbohydrate diet groups, the LCHF diet resulted in a more pronounced effect on the gut microbiota increasing the relative abundance of bacterial taxa with recognized capabilities for lipid metabolism. The relative abundance of *Bacteroides* spp. was negatively correlated with fat oxidation and the relative abundance of *Dorea* was negatively correlated with an exercise economy test. It appears that individual responsiveness to a high-fat diet may affect the amount of dietary fat that actually reaches the distal gut, where it could have associative effects on the gut microbiota. Furthermore, relative abundance of *Faecalibacterium* spp. was decreased in athletes after consumption of the LCHF diet. Interestingly, *Faecalibacterium* spp. is one of the most abundant bacterial taxa present in the gut microbiota of healthy individuals and has been linked to a host of metabolic products with anti-inflammatory effects [231]. Diets high in fat likely increase the pool of bile acids that elude epithelial absorption in the GI tract and interact with the gut microbiota [232]. This interaction can impact the composition of the gut microbiota including reductions in the relative abundance of *Faecalibacterium* spp. [233]. *Faecalibacterium* is widely recognized for its production of a suite of metabolites and peptides with anti-inflammatory effects [231].

#### Summary of the effect of athletic diet on the gut microbiota

Overall, there is a need for longer-term studies in different athletic cohorts examining the impact of diet on the structure and function of the gut microbiota. This approach is of particular importance as many athletes follow special dietary practices, such as during periods of intensified training prior to competition and offseason periods. Research studies should investigate exercise-nutrient interactions that underpin adaptation and performance [234]. Finally, further research is needed to determine the synthesis kinetics and clinical consequence of microbial by-products during increased nutritional status and metabolic demands during exercise. Ultimately modulation of the microbiota and its fermentation capacity may be considered in dietary prescription for athletes. This may include specific nutrient recommendations aimed at improving performance by enhancing certain metabolites during exercise and recovery, and limiting those that produce toxic metabolites that may made worsen the consequences of exercise stress [15].

**Table:** 

**Key Points 3 – Influence of Athletic Diet on Gut Microbiota.**	
• Diet is an established modulator of gut microbiota composition and activity, with marked changes in microbiota composition evident within 24 h of a dietary change.	
• Energy balance is currently an overlooked factor in relation to the athletic gut microbiota, particularly in those affected by RED-S.	
• To solely investigate the impact of total energy consumption without considering dietary variability is difficult (if not impossible).	
• The effects of high-protein consumption (without concurrent high-fat) on gut bacteria are not well studied. This also includes the effects of high-protein and high-fiber intake.	
• Protein intake appears to be a strong modulator of microbiota diversity, with protein supplementation, such as whey, showing potential benefits that need further study in humans.	
• Proteins from vegetable origin have a marked effect on gut microbiota but currently require investigation in athletes.	
• In future studies, the types and amounts of fats consumed in conjunction with protein should be investigated in the overall effect on the gut microbiota.	
• Increased intake of dietary fiber is associated with microbial richness and/or diversity.	
• Higher intake of carbohydrate and dietary fiber in athletes appear to be associated with increased abundance of *Prevotella*.	
• The specific effects of fat on the gut microbiota is difficult to isolate; however, the types of fats consumed appear to be important.	

## Conclusion

The current body of literature, although limited, indicates that the cluster of athletic components such as exercise, associated dietary factors, and body composition promotes a more “health-associated” gut microbiota. Typical features include a higher abundance of health-promoting bacterial species, increased microbial diversity, functional pathways, and microbial-associated metabolites, stimulation of bacterial abundance that can modulate mucosal immunity, and improved barrier functions. In comparison to sedentary controls, athletes have increased fecal metabolites and improved overall health (unless over-trained or in RED-S). However, in sedentary individuals, exercise appears to positively modulate the composition and metabolic capacity of the human gut microbiota. Given that athletes generally have a distinct diet, research on the gut microbiome in athletes must incorporate dietary and supplemental intake otherwise it might be a confounding factor in determining exercise-specific effects on the composition of the microbiome. While individuals’ microbiotas appear to be driven by their primary dietary patterns, future research is needed to better describe the impact of high-protein consumption and (in conjunction) the types and amount of fiber and fats consumed. Investigators should examine how different types of sport, athlete, and physical training regimens influence the gut microbiota. The present review focuses on the discussion of the results from microbiota-related studies, however, a deep discussion of the methodological approaches of each manuscript was not possible due to the already extended content. Future, more specific reviews in this research area should aim for discussing the results in the frame of their methodological approaches. Finally, much of the current research is cross-sectional and has relied on 16S rRNA sequencing. Therefore, future research should employ longitudinal designs as well as more advanced high-throughput sequencing and bioinformatic analyses to provide deeper understanding and functional causation of the gut microbial influence on athlete health and performance. This information can then be used to develop novel therapeutic and nutritional strategies to modulate the microbiota and enhance the athlete’s overall performance and health. Ultimately this body of work will define how metabolic capabilities of gut microbiota are shaped by exercise and elucidate their functional roles influencing health and disease.

## Data Availability

Not applicable.

## Abbreviations

BCAA: Branched-chain amino acid
BMI: Body mass index
GI: Gastrointestinal
Kcal: Kilocalories
LCHF: Low-carbohydrate, high-fat
LPS: Lipopolysaccharide
OTUs: Operational taxonomic units
RED-S: Relative Energy Deficiency in Sports
rRNA: Ribosomal RNA
SFCA: Short chain fatty acid
Spp.: Species
VO_2_: Volume of oxygen utilization
